# Mobile Apps for the Personal Safety of At-Risk Children and Youth: Scoping Review

**DOI:** 10.2196/58127

**Published:** 2024-11-05

**Authors:** Camille Bowen-Forbes, Tilovatul Khondaker, Tania Stafinski, Maliheh Hadizadeh, Devidas Menon

**Affiliations:** 1 Health Technology and Policy Unit School of Public Health Edmonton, AB Canada

**Keywords:** children, youth, personal safety apps, smartphones, mobile apps, violence, bullying, suicide prevention, youth support, homeless support, mobile phone

## Abstract

**Background:**

Personal safety is a widespread public health issue that affects people of all demographics. There is a growing interest in the use of mobile apps for enhancing personal safety, particularly for children and youth at risk, who are among the most vulnerable groups in society.

**Objective:**

This study aims to explore what is known about the use of mobile apps for personal safety among children and youth identified to be “at risk.”

**Methods:**

A scoping review following published methodological guidelines was conducted. In total, 5 databases (Scopus, SocINDEX, PsycINFO, Compendex, and Inspec Archive) were searched for relevant scholarly articles published between January 2005 and October 2023. The gray literature was searched using Google and Google Scholar search engines. The results were reported using the PRISMA-ScR (Preferred Reporting Items for Systematic Reviews and Meta-Analyses Extension for Scoping Reviews) guidelines. For summarizing the features and users’ experiences of the apps, a published framework for evaluating the quality of mobile health apps for youth was used.

**Results:**

A total of 1986 articles were identified, and 41 (2.1%) were included in the review. Nine personal safety apps were captured and categorized into 4 groups based on the goals of the apps, as follows: dating and sexual violence prevention (n=4, 44% of apps), bullying and school violence prevention (n=2, 22% of apps), self-harm and suicide prevention (n=2, 22% of apps), and homeless youth support (n=1, 11% of apps). Of the 41 articles, 25 (61%) provided data solely on app descriptions and features, while the remaining 16 (39%) articles provided data on app evaluations and descriptions. Outcomes focused on app engagement, users’ experiences, and effectiveness. Four articles reported on app use, 3 (75%) of which reported relatively high app use. Data on users’ experience were obtained from 13 studies. In general, participants found the app features to be easy to use and useful as educational resources and personal safety tools. Most of the views were positive. Negative perceptions included redundancy of app features and a lack of usefulness. Five apps were evaluated for effectiveness (n=2, 40% dating and sexual violence prevention; n=2, 40% self-harm and suicide prevention; and n=1, 20% bullying and school violence prevention) and were all associated with a statistically significant reduction (*P*=.001 to .048) in harm or risk to participants at the 95% CI.

**Conclusions:**

Although many personal safety apps are available, few studies have specifically evaluated those designed for youth. However, the evidence suggests that mobile safety apps generally appear to be beneficial for reducing harm to at-risk children and youth without any associated adverse events. Recommendations for future research have been made to strengthen the evidence and increase the availability of effective personal safety apps for children and youth.

## Introduction

### Background

Interpersonal violence is a global public health and human rights challenge, having effects at the family, community, and national levels, with impacts reverberating across generations [1]. Physical violence, psychological violence, verbal abuse, and sexual assault or harassment are common forms of interpersonal violence [2]. More specific examples are child abuse, dating violence, domestic violence, human trafficking, stalking, hazing, bullying, and older person abuse [3]. It is a leading cause of not only physical and psychological harm but also early mortality and is therefore a significant threat to personal safety [1,4]. Interpersonal violence, therefore, has considerable societal consequences, including significant economic burden due to health care provision, loss of productivity, criminal justice involvement, and antiviolence campaigns and interventions [4,5]. The total economic cost of violence has been estimated to be in the billions for many countries worldwide, including Canada [6,7], the United States [8], and the United Kingdom [5,9].

While every age demographic experiences interpersonal violence, children and youth constitute a particularly important sector. This is because such violence, which can reoccur throughout an individual’s life, has enduring consequences, increasing one’s lifelong vulnerability to a myriad of emotional and physical health problems and negative health behaviors such as substance misuse and risky sexual behaviors [1,10,11]. In 2020, it was estimated that 1 billion children (1 out of every 2 children worldwide) experience some form of violence each year. When aggregated across billions of people, the effects of violence against children can have detrimental effects on economic development [12]. It has been reported that individuals between 12 and 34 years of age are at the highest risk for sexual assault [13]. In a study involving 8629 participants in the United States, violent childhood experiences were reported to double the risk of experiencing intimate partner violence (IPV) in adulthood for women and double the risk of IPV perpetration in men [14]. Studies such as this show that the impacts on younger populations can be more devastating, affecting individuals, families, communities, and society as a whole. It has been shown that children and youth are among the sectors of society that are at greatest risk of violence, sexual abuse, and going missing [15,16]. Among other factors, youth and young adults are at increased risk of victimization, as they are more likely to be single, have lower income, and engage to greater extents in nighttime activities [4].

In addition to harm from older adults, children and youth are also at risk from their peers through incidents such as dating violence, sexual violence, and bullying [10,17,18]. A Youth Risk Behavior Survey conducted in 2019 among high school students in the United States revealed that 25% of students reported bullying victimization and approximately 12% reported dating violence (physical or sexual) [10]. Furthermore, interpersonal violence is one of the main causes of death among adolescents and young adults in most countries worldwide [1].

Youth personal safety is not only impacted by interpersonal violence but also by the risk of self-harm. Mental health issues such as depression, anxiety, and suicide pose significant threats to the lives and well-being of individuals between 10 and 24 years of age worldwide [19]. The World Health Organization describes children as persons aged <18 years and youth as persons between 15 and 24 years of age [19,20]. With people aged <25 years accounting for 42% of the world population and being among the most vulnerable [21], measures to protect their lives and well-being are of utmost importance.

Addressing the issue of violence requires a multifaceted approach involving various levels of society. Mobile technology greatly expands the possible range of available options for addressing these issues [22]. Increasingly, health and human service organizations, policy makers, as well as practitioners across the world have recognized the potential of smartphone apps in helping to address social issues including interpersonal violence and mental health issues at both individual and community levels [5]. The global increase in smartphone ownership makes this option even more potentially useful. The number of smartphone users worldwide has continuously increased from approximately 1 billion in 2014 to 4.88 billion in 2024 and is forecast to reach 6.4 billion by 2029 [23]. There were almost 7 billion smartphone mobile network subscriptions worldwide in 2023, and this number is expected to exceed 7.7 billion by 2028 [24]. Smartphone apps are a particularly important avenue for addressing youth’s issues, as youth tend to be more open to technological services compared to the more traditional approaches [25]. The prevalence of smartphones among the younger demographic is well known [26], with smartphones being ubiquitous among youth and young adults. As of January 2024, a significant 98% of Gen Z (people born between 1997 and 2012) own a smartphone [27]. In April 2022, a significant 87% of teenagers between 12 and 17 years of age in Canada were using smartphones; half of the children between 7 and 11 years of age and 39% of children between 2 and 6 years of age were reported to use a mobile device [28]. Similarly, in the United States, 88% of teenagers aged between 13 and 18 years owned a smartphone in 2021; among younger kids between 8 and 12 years of age, ownership of tablets (57%) was more prevalent than smartphone ownership (43%) [29].

Widespread access to mobile phones opens up opportunities for their use as tools to mitigate the risk of harm to children and youth, improving outcomes in instances when such incidences occur. There has been a growing interest in the use of mobile apps for enhancing personal safety; however, there is a lack of evidence on the use and effectiveness of such apps that are specifically geared toward protecting children and youth. Most of the literature on mobile apps has been focused on health and fitness [4,30-38]. There are also some studies on personal safety apps, but most of them either cover a wide age demographic, are focused on sexual violence against women (with no youth focus), or are focused solely on app development with no associated evaluation [5,39-43]. Furthermore, evidence on apps intended for autonomous use by children and youth is lacking in the literature. Ford et al [5] published an overview of smartphone apps available in the United Kingdom. Of the 86 apps included in the study, 52% targeted the general population, 26% targeted women, and 13% targeted families. None of the studies specifically targeted youth. Nonetheless, that research found that app functionality included providing an alarm (22%), sending alerts to predesignated contacts (71%), providing evidence capture (34%), and offering educational information (26%). More than 70% of apps had a user rating of at least 4 out of 5. Key aspects included positive consequences of app use, technical issues, dissatisfaction with the financial cost of some features, and ethical issues [5]. The effectiveness of the apps was not evaluated.

Most of the literature on personal safety apps is focused on preventing sexual violence or domestic violence, particularly against women. This is not surprising, considering the high prevalence of sexual violence victimization in women globally. For example, in Canada, the rate of IPV was >3 times higher among women and girls compared to men and boys in 2022 [15]. Doria et al [39] identified 3 themes in their review on women’s experience with safety apps: security, accessibility, and knowledge. Although there was no evaluation of effectiveness, a common thread among most of the app users was their view that the apps were acceptable, user-friendly, and useful [15]. The review highlighted the potential of smartphone interventions to become a valuable tool for preventing sexual violence in women. Sumra et al [44] conducted a systematic review that included 136 smartphone apps that targeted domestic violence prevention. They found that over two-thirds of the apps (71%) were released between 2020 and 2022, with almost a half of them (46%) being from northeast America. Five app categories were described: emergency assistance (44%), avoidance (21%), informative (21%), legal information (7%), and self-assessment (5%) [44], which were similar to those identified by other researchers [45]. Unique features among the apps included geo-fences, shake-based alert, accelerometer-based alert, alert auto cancelation, anonymous communication, and data encryption [44]. None of the apps had automated alerts or used artificial intelligence to help potential survivors. There was no focus on youth and no evaluation of effectiveness. A 2016 systematic app search for intimate partner and sexual violence prevention and response apps found that, of the 132 unique apps identified, 66% targeted adults, 24% targeted the general population, 27% targeted young adults, 10% targeted teens, and 2% targeted children aged <12 years. However, the app categories were not mutually exclusive, and the specific apps were not identified. As a result, it is impossible to determine what proportion of the apps specifically targeted the younger demographic or to identify them [13]. The apps were found to vary greatly in quality, and sharing information or resources was the primary purpose of most of the apps (76%).

Draughon Moret et al [13], who were experienced forensic examiners, reported that there were only a few apps that they would use as clinicians or recommend to their patients after a physical or sexual assault. The apps focused largely on education and information sharing; therefore, it was thought that they may not successfully meet their desired goal. In addition, they experienced difficulty in finding the apps, as searches for violence prevention and response apps yielded many disturbing apps (zombie-killing games, dating sims, etc), which could potentially retraumatize patients. Furthermore, there was a lack of quality and evidence base among the apps [13].

Reviews focused on sexual violence or domestic violence prevention have found that most of the apps addressed emergencies, with a large proportion of apps focusing on avoidance or education [44-46]. They concluded that further research on app development should focus on automation, making better use of artificial intelligence, speech recognition, and pitch detection to assist in live analysis of the situation and for accurately generating emergency alerts [44]. Other recommendations for further research include a greater focus on app efficacy, sustainability, and data security [45].

Despite widespread access to mobile apps and the growing interest in their use for enhancing personal safety, there is a lack of evidence on the use and effectiveness of such apps that are specifically geared toward protecting children and youth.

### Objective

This review aimed to understand what is known about the use of mobile apps for personal safety among at-risk children and youth.

## Methods

### Overview

A scoping review was conducted following published methodological guidelines by Arksey and O’Malley [47]. They comprise the following 6 steps: identifying the research question; consulting with stakeholders (an optional step in the framework); identifying relevant studies; selecting studies; charting the data; and collating, summarizing, and reporting the results [47]. A scoping review was conducted, as this type of review is particularly useful for mapping the scope, range, and character of the literature and identifying any potential gaps in the body of knowledge on a given topic [48]. Multimedia Appendix 1 [49] provides the PRISMA-ScR (Preferred Reporting Items for Systematic Reviews and Meta-Analyses Extension for Scoping Reviews) checklist [49]. No protocol for this review was previously published. The term “at-risk children and youth” refers to those who are in physical or mental danger [50].

The main categories of the theoretical framework developed by Jeminiwa et al [51] for evaluating the quality of mobile health (mHealth) apps for adolescent users were used to provide an overview of app features. The framework has 5 categories (technical quality; engagement; support system; autonomy; and safety, privacy, and trust). However, the authors of this paper modified it to include “esthetics” (included as a subcategory of “engagement” in the framework by Jeminiwa et al [51]) as a distinct category to cover layout, graphics, and visual appeal. In addition, a “subjective quality” category was added to cover concepts such as usefulness and recommendability. The modifications were guided by the features of the validated Mobile App Rating Scale [52,53], and “personal safety” was also added by the authors to capture features such as self-tracking and a panic button (Multimedia Appendix 2 [51,52]).

### Identifying the Research Question

The research question was as follows: “What is known about the use of mobile apps to ensure personal safety among at-risk children and youth?”

### Consulting With Stakeholders

To inform the research, a police service division in Alberta planning to develop a personal safety app for at-risk children and youth provided information on important issues to consider. Topics such as app features, use, users’ perceptions, and effectiveness were discussed. Issues related to privacy and security were also discussed. A computing science professor from the University of Saskatchewan with expertise in the development of apps also offered insights into key aspects of personal safety apps for youth.

### Identifying Relevant Studies

Both peer-reviewed and gray literature sources were included in this review. As non–peer-reviewed sources (eg, reports and app-specific websites) can provide valuable insights and perspectives that may not be captured solely through peer-reviewed literature, these sources were included. In particular, they provided useful information on the characteristics of the various apps. With support from an experienced research librarian, a search strategy for scholarly literature was developed and tested iteratively. In total, 5 databases were searched: Scopus, SocIndex (EBSCO platform), PsycINFO (Ovid platform), Compendex, and Inspec Archive (both Engineering Village platforms). The searches were performed from July 19 to July 30, 2023, using combinations of relevant terms, such as “at-risk,” “youth,” “children,” “safety,” and “mobile application.” Keywords included “homeless teenagers,” “runaway children,” “abandoned children,” “street youth,” “school-aged,” “Indigenous youth,” “poor children,” “juvenile offenders,” “LGBTQ+,” “sexually abused teenagers,” “domestic violence,” “protect,” “prevent,” “safety app,” “mobile-based,” and “smartphone.” Adjustments to the search strategy across different databases were made due to database-specific indexing or features. For example, both APA PsycINFO (Ovid platform) and SocINDEX (Ebsco platform) include extensive but differing controlled vocabularies for children who are abused, fostered, homeless, or neglected and their care. APA PsycINFO uses terms such as “foster care,” “child neglect,” and “protective services,” whereas SocINDEX uses “foster home care,” “child abuse,” and “child protection services.” Where possible, equivalent free-text terms were used across all the databases. Syntax was adjusted according to the specifications of each database or platform. All citations were imported into EndNote version 9.3.3 (Clarivate Analytics, Inc), and duplicates were removed. For the gray literature, Google and Google Scholar were searched using similar terms to those applied to the peer-reviewed strategy.

### Study Selection

Included articles satisfied the following criteria: (1) participants were at-risk children or youth; (2) the article focused on mobile apps designed for personal safety; (3) the children and youth had autonomous control of the app; (4) the article was published between 2005 and 2023, as the use of mobile apps for safety applications has been fairly recent; and (5) the evaluation study assessed app users’ experience, app engagement, or app impact. Articles intended to be used for characterizing the apps did not need to be evaluation studies. As the terms “children” and “youth” are variously described in the literature, no strict age limits were applied for inclusion; rather, if the target or study population was described using descriptors for children and youth such as “teenagers,” “adolescents,” or “college students,” the study was included. Due to the paucity of available articles, studies focused on participants not strictly considered “at risk” were also included, as long as they focused on children and youth. If ≥1 of the abovementioned criteria were not satisfied for a given app, the articles were excluded. Bullying prevention apps focusing solely on cyberbullying were outside of the scope of this project and were therefore excluded, as were articles not available in English.

For the peer-reviewed literature search, 2 researchers (CB-F and TK) independently screened the titles and abstracts of the identified articles. For quality assurance, a portion of the articles was reviewed by both researchers. Conflicts were resolved through discussion. In cases of disagreement, a third researcher (DM) arbitrated. For the gray literature search, the same 2 researchers systematically searched Google and Google Scholar using similar keywords to those applied to the peer-reviewed search and scanned the first 50 “hits” generated from applying the search terms. Excel software (Microsoft Corporation) was used for data management.

### Charting the Data

Information collected from papers was extracted using a standard template. The data extracted included the following elements: goal of app, operating system, date launched, provider or developer, target users, general description, features, app funding, study aim, study type, study period, methods, participants, outcomes measured, findings, facilitators and barriers to app use, app limitations, and conclusions and recommendations. The data extraction tables were piloted and revised as necessary. To ensure consistency in data extraction, CB-F and TK each independently extracted data from a single article and then reviewed each other’s work to establish a consistent approach to charting. The researchers met several times during the screening process to ensure a consistent data charting approach.

### Collating, Summarizing, and Reporting the Results

The apps were categorized into 4 groups based on app goals and target populations. A descriptive analytical approach was then used to summarize the findings. This involved using common analytical frameworks for summarizing different aspects of the included articles and collecting standard information from each of them [47]. For example, to summarize app features and users’ perceptions, a modified version of the framework developed by Jeminiwa et al [51] was used. For app features, 6 categories were captured: engagement; esthetics; support system; personal safety; autonomy; and safety, privacy, and trust. Four categories were captured for users’ perception: engagement; esthetics; safety, privacy, and trust; and subjective quality. Evidence on the effectiveness of the apps was organized by outcomes, such as IPV and other sexual violence, school violence and bullying, and suicide ideation and suicide risk. Data on app evaluation were summarized in Microsoft Excel spreadsheets.

## Results

### Results of Literature Search

**Figure 1:**
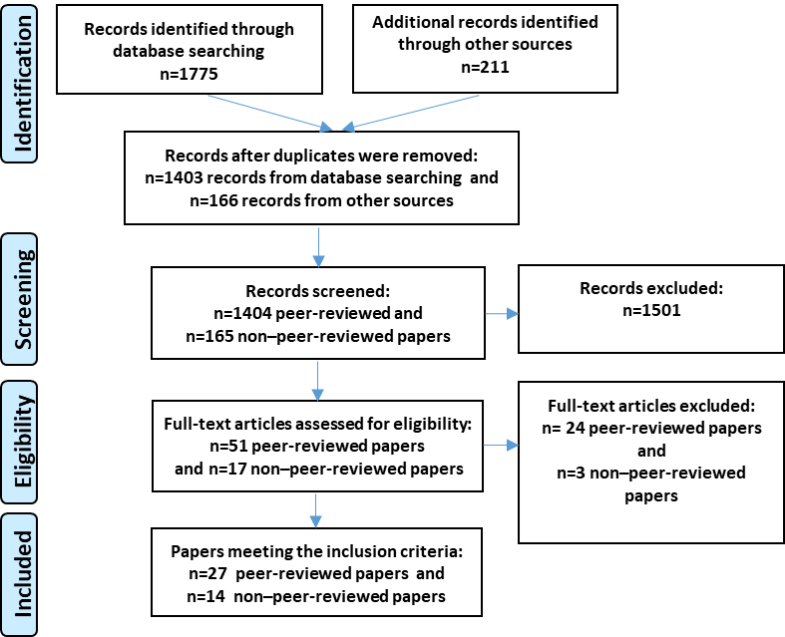
PRISMA (Preferred Reporting Item for Systematic Reviews and Meta-Analyses) flow diagram of study selection. Additional records identified through other sources include both non–peer-reviewed and peer-reviewed articles. Reasons for exclusion of studies include the following: not focused on children or youth; not focused on personal safety apps; no information on app use, users’ perception, or app impact; published before 2005; and non-English.

A total of 1986 articles were identified through peer-reviewed (n=1775, 89.37% articles) and gray literature searches (n=211, 10.62% articles). Most of the identified literature on mobile apps primarily focused on health and fitness [4,30-37]. There were also some studies on personal safety apps; however, most either covered a broad or older demographic, focused on sexual violence against women, or solely addressed app development without evaluation [5,38-42]. Consequently, of the 1986 articles, only 68 (3.42%) were eligible for full-text screening. Finally, 41 articles (n=27, 66% peer-reviewed and n=14, 34% non–peer-reviewed) met the inclusion criteria and were included for data extraction. Collectively, these 41 articles provided data on the features and evaluation of 9 apps that met our inclusion criteria. Several studies reported on various aspects or phases of the app development and evaluation process in different articles. For example, for 1 app, acceptability and impact were captured in 2 separate articles [54,55]. The non–peer-reviewed literature primarily provided detailed information on app characterization, including descriptions, features, and functionalities. The results of the screening and selection process are presented in the PRISMA (Preferred Reporting Items for Systematic Reviews and Meta-Analyses) flow diagram (Figure 1).

### Basis of App Development and Stakeholder Engagement

All 9 apps identified through the included articles are summarized in Table 1, and further details are provided in Multimedia Appendix 3 [54-91]. In addition to the language used in the respective articles to describe the type of app, the descriptors from the typology of crime prevention apps by Wood et al [56] were also used. For example, some apps were described as decision aid apps, which are apps designed to help individuals in making decisions based on high-quality evidence [56]. The use of a relevant theory as a basis for development was noted for 3 (33%) of the 9 apps: ambivalent sexism theory and romantic love myths for Liad@s (Universitat de València) [57,58], cognitive behavior theory and dialectic behavior therapy for BlueIce (Oxford Health NHS), and acceptance and commitment therapy for iBobbly (Black Dog Institute). In addition, myPlan (Johns Hopkins University School of Nursing) was developed based on literature on empowerment, internet safety decision aid, and safety planning [59]; +FORT (AXEL; University of Montréal) was developed based on a coordinated sequence of 4 mechanisms of action central to problem-solving [60]; and organizations focused on domestic and sexual violence were consulted in the development of uSafeUS (University of New Hampshire) [61]. The development of the YTH Street Connect prototype app (Santa Clara University Frugal Innovation Hub) was based on information obtained from formative research on homeless or unstably housed youth and mHealth apps and in consultation with homeless or unstably housed service providers [62]. Collaborators from multiple sectors were involved in the development of most of the included apps (7/9, 77%). They included universities (7/9, 77%), schools (2/9, 22%), target users (7/9, 77%), parents (3/9, 33%), companies or organizations (3/9, 33%), and mental health professionals (2/9, 22%). In total, 7 (77%) of the 9 apps are available in English only, while 1 (11%) is available in English and French (+FORT) and 1 (11%) is available in Spanish (Liad@s). In total, 6 (66%) of the 9 apps are currently available, 5 (56%) of which may be freely downloaded and 1 app, 1 (11%) is available only by prescription from child mental health services [54,55]. Of the 3 apps that are not currently available, 2 (67%) were simply prototype apps (YTH StreetConnect) [62] and Circle of 6 [Co6] (Youth Tech Health; Kliq) [63]) and 1 (33%; iBobbly) was recently discontinued [92]. On the basis of an official statement of the First Nations team at the Black Dog Institute (iBobbly developers) in New South Wales, Australia, iBobbly was decommissioned in response to evolving community needs, with the focus now being on providing and recommending best-in-market products (email, November 12, 2023).

**Table 1:** Overview of app features.

App features and descriptors			Homeless youth support		Dating and sexual violence prevention									Bullying and school violence prevention					Self-harm and suicide prevention		
			YTH StreetConnect [62]		Circle of 6 [63]		Liad@s [57,64]		myPlan [59,65-67]		uSafeUS [61,68,93]		+FORT [60,69,91]			uSafeHS [70,71]		BlueIce [54, 55,72,73]			iBobbly [74-78]
**Engagement**																					
	Customizable features							✓		✓					✓		✓			✓	
	Activities for youth					✓											✓			✓	
	Gamified					✓									✓						
**Aesthetics**																					
	Appealing design	✓						✓				✓			✓		✓			✓	
**Support system**																					
	Educational content	✓		✓		✓		✓		✓		✓			✓		✓			✓	
	Resource locator	✓								✓					✓						
	Decision aid or personalized action plan	✓						✓		✓										✓	
	Youth and admin platforms	✓													✓		✓				
	Diary or self-checks											✓					✓			✓	
	Trusted contacts	✓		✓													✓				
**Personal safety**																					
	Panic button									✓											
	Self-tracking	✓		✓						✓											
	Fake call or text			✓						✓											
	Emergency call or text	✓		✓				✓									✓			✓	
	Incident reporting									✓					✓						
**Autonomy**																					
	Youth controlled	✓		✓		✓		✓		✓		✓			✓		✓			✓	
	Free					✓		✓		✓		✓									
**Safety, privacy, and trust**																					
	Android and iOS	✓		✓		✓		✓		✓		✓			✓		✓			✓	
	PIN^a^ or password protected							✓		✓					✓		✓			✓	

^a^PIN: personal identification number.

### App Characteristics

The apps were categorized into 4 groups based on app goals and target populations: homeless youth support (1 app), dating and sexual violence prevention (4 apps), bullying and school violence prevention (1 app), and self-harm and suicide prevention (2 apps). A more detailed description is provided in Multimedia Appendix 3. All 9 apps were designed for the autonomous use of youth. They all featured goal-specific educational content and were available on both iOS and Android devices.

### Homeless Youth Support

YTH StreetConnect is a decision aid mobile phone app developed in 2016 with the goal of connecting homeless or unstably housed youth in Santa Clara County, California, United States, to health and vital resources [62]. YTH StreetConnect Pro is a companion tablet app for providers who serve these youth [62]. Both are discussed as a single app for the purpose of this review. YTH StreetConnect has features such as a location-based database, interactive mapping, and emergency hotlines. The app helps youth locate services using visual enhancements. Youth have access to sexual health information and weekly health tips via SMS text messaging. YTH StreetConnect Pro features include a referral function and a medical questionnaire to assess clients’ homelessness vulnerability and sexual risk [62].

### Dating and Sexual Violence Prevention

Four dating and sexual violence prevention apps were identified, 3 (75%) of which were developed in the United States (Co6, myPlan, and USafeUS) and 1 (Liad@s) in Spain [57,61,63,66-68,79,93]. Liad@s targets adolescents, USafeUS targets college or university students, and Co6 and myPlan target people at risk of sexual violence. Two of the apps have decision aid or personalized action plan features (myPlan and uSafeUS), and 1 app has an interactive game format (Liad@s). Two apps have an emergency text or a fake-a-call or text feature (Co6 and USafeUS, respectively), and 1 (USafeUS) app has an incident reporting feature. Co6, which was a pilot app, is not currently available on the market [80].

### Bullying and School Violence Prevention

Two apps were identified in the bullying and school violence prevention category: +FORT (Canada) [60,69,81] and uSafeHS (University of New Hampshire; United States) [70,71]. +FORT, developed in Quebec, was first available in French and was subsequently made available in English. Both apps target high school students, with +FORT aimed at preventing bullying victimization. uSafeHS aims to prevent school violence in general, including bullying (Multimedia Appendix 3). +FORT allows youth to journal their bullying victimization experiences and compiles the information in simple graphs, which youth may use to enhance their safety awareness [60,81]. uSafeHS has an administrative platform, incident reporting, and an interactive game feature to facilitate social and emotional learning [70,71].

### Self-Harm and Suicide Prevention

BlueIce [54,55,72,73,82-85] and iBobbly [74-78,86,87] are self-harm and suicide prevention apps that were identified. BlueIce targets young people attending Child and Adolescent Mental Health Services across the United Kingdom and aims to help them reduce urges to self-harm. iBobbly targeted Aboriginal and Torres Strait Islander Australians aged ≥15 years (youth, in particular) and aimed to tackle suicide prevention in a culturally appropriate way. Both have emergency call or text, diary or self-check, and activities that youth may engage in as features.

### Characteristics of App Evaluation Studies

A total of 14 studies conducted between 2013 and 2022 were identified and reported in 16 peer-reviewed papers. In total, 15 papers reported on experimental study designs (3 randomized controlled trials [RCTs] [59,74,76], 9 single-arm design [54,55,61-63,65,66,70,79], 2 quasi-experimental design [57,64], and 1 that used single-arm and quasi-experimental designs in 2 phases [60]). One paper reported on a cross-sectional study [93]. All the studies included self-reported data. The studies were conducted in the United States (5 apps and 9 papers [59,61-63,65,66,70,79,93]), Australia [74,76], Canada [60], Spain [57,64], and the United Kingdom [54,55] (1 app each).

The 14 studies were conducted across several different settings as follows: 6 (43%) in college or universities [59,61,63,65,66,79,93]; 3 (21%) in high schools [60,70], and 1 (7%) each in mental health services for children [54,55], childcare homes [57], Aboriginal and Torres Strait Islander communities [74,76], the homeless youth context [62], and dating locations [63].

The duration of app use ranged from 20 to 40 minutes in 1 single-arm qualitative study [65] to 12 months in an RCT. For 7 (50%) of the 14 studies (corresponding to 6 apps), the apps were used for at least 6 weeks.

### Characteristics of Participants

The reviewed studies included >3800 children and youth, but demographics were not consistently reported. Reported average age ranged from 14 to 27 years; in 1 study, one-third were aged ≥26 years [93], and in another study, the age range was 19 to 29 years [74]. Where information on sex was reported, only 1 study had more men than women [57] (in a childcare home), and in 2 studies evaluating dating and sexual violence prevention apps, only women were enrolled [59,63]. Only 3 studies captured information on sexual orientation [62,63,79]. Where reported, the proportion of White participants ranged from 33% (2/6) [62] to 71.3% (122/171) [59,62].

In addition to children and youth, other stakeholders (eg, parents, app administrators, and police officers) were included as participants in the evaluation of 4 apps [54,55,60,61,70].

### Study Outcomes and Measurement Approaches

Table 2 provides a summary of the outcomes and associated measurement tools used in the included studies. Outcomes comprised app engagement (4/9, 44% apps), users’ experiences (9/9, 100% apps), and effectiveness of the app (7/9, 78% apps; Multimedia Appendix 4 [54,55,57,59-66,70,74,76,79,92,93]). Effect measures were safety behavior, bystander behavior, sexism, IPV, self-harm, suicide risk, depression, anxiety, and bullying victimization. A wide range of tools was used for measuring outcomes. Self-reported measures were used for all 3 types of outcomes reported (app use: 2/4, 50% apps, users’ experience: 9/9, 100% apps, and effectiveness: 7/7, 100% apps). Validated tools were used in the evaluation of effectiveness of 86% (6/7) of the apps. For the remaining app (+FORT), even though no validated tool was used for measuring effectiveness, the app itself uses items adapted from the validated Olweus Bullying Questionnaire for logging information about users’ bullying experiences to help users identify more effective strategies to deal with bullying victimization [60]. Objective measurements were used for measuring app use for 2 (50%) out of 4 apps and for measuring the effectiveness of 1 (14%) out of 7 apps. For example, for the BlueIce app, the change in users’ frequency of self-harming was determined by comparing historical clinical data with the self-reports of postintervention self-harming incidence [46]. For 3 apps, the general internet use of app users was also assessed, and self-reported, unvalidated measures were used [54,55,62,70]. Further details on study outcomes and measurement approaches are provided in Multimedia Appendix 4.

**Table 2:** Study outcomes and measurement approaches.

Outcomes and tool name and/or description			Mode of measurement		App		Study
**General internet use**							
	Question, questionnaire, survey, or interview	Self-reported and unvalidated		iBobbly, uSafeHS, and YTH StreetConnect		[62,70, 74]	
**App engagement (downloads, frequency of use, features used, etc)**							
	Mobile device download data	Objectively measured		iBobbly		[74]	
	Administrative dashboard analytics	Objectively measured		usSafeHS		[70]	
	Question, questionnaire, survey, or interview	Self-reported and unvalidated		Circle of 6		[63]	
	Question, questionnaire, survey, or interview	Self-reported and unvalidated		uSafeUS		[93]	
**Users’ experience (perception, feasibility, acceptability, or subjective value)**							
	Question, questionnaire, survey, or interview	Self-reported and unvalidated		BlueIce, Circle of 6, +FORT, Liad@s, iBobbly, myPlan, uSafeHS, uSafeUS, and YTH StreetConnect		[54,57, 59-63, 65,66, 70,74, 79,93]	
**Effectiveness**							
	Decisional conflict: Decisional Conflict Scale (modified)	Self-reported and validated		myPlan		[59]	
	Safety behaviors: question, questionnaire, survey, or interview (number of safety behaviors tried on app)	Self-reported and validated		myPlan		[59]	
	Bystander behavior: Banyard’s Bystander Scale	Self-reported and validated		Circle of 6		[63]	
	Intention to help: 10-item modified Intention to Help Scale	Self-reported and validated		Circle of 6		[63]	
	Sexism (hostile sexism and benevolent sexism): Ambivalent Sexism Inventory-Adolescents	Self-reported and validated		Liad@s		[57,64]	
	Ambivalence toward men: Ambivalence toward Men Inventory	Self-reported and validated		Liad@s		[57]	
	Distortions about romantic love: Myths, Fallacies, and Erroneous Beliefs about the Ideal of Romantic Love Scale	Self-reported and validated		Liad@s		[57]	
	Sexual victimization: 11 item-Revised Sexual Experience Survey	Self-reported and validated		Circle of 6		[63]	
	Intimate partner violence: Composite Abuse Scale	Self-reported and validated		myPlan		[59]	
	Intimate partner violence: traumatic brain injury (questions or questionnaire)	Self-reported and unvalidated		Liad@s		[57]	
	Intimate partner violence: digital abuse (questions or questionnaire)	Self-reported and unvalidated		myPlan		[59]	
	Intimate partner violence: reproductive coercion (questions or questionnaire)	Self-reported and unvalidated		myPlan		[59]	
	Substance use: frequency of alcohol use	Self-reported and unvalidated		Circle of 6 and myPlan		[59,63]	
	Substance use: frequency of getting drunk	Self-reported and unvalidated		myPlan		[59]	
	Substance use: frequency of binge drinking	Self-reported and unvalidated		myPlan		[59]	
	Substance use: any marijuana use	Self-reported and unvalidated		myPlan		[59]	
	Substance use: any drug use other than marijuana	Self-reported and unvalidated		myPlan		[59]	
	Substance use: feeling of intoxication	Self-reported and unvalidated		Circle of 6		[63]	
	Self-harm: clinical data	Objectively measured		BlueIce		[63]	
	Self-harm: question, questionnaire, survey, and interview	Self-reported and unvalidated		BlueIce		[63]	
	Bullying victimization: Multidimensional Peer-Victimization Scale	Self-reported and validated		+FORT		[60]	
	Depression: Center for Epidemiologic Studies Depression Scale Revised	Self-reported and validated		myPlan		[59]	
	Depression: Mood and Feelings Questionnaire	Self-reported and validated		BlueIce		[54,55]	
	Depression: Patient Health Questionnaire 9	Self-reported and validated		iBobbly		[74,76]	
	Anxiety: Revised Child Anxiety and Depression Score	Self-reported and validated		BlueIce		[54,55]	
	Psychological distress: Kessler Psychological Distress Scale	Self-reported and validated		iBobbly		[74,76]	
	Impulsivity: Barratt Impulsivity Scale	Self-reported and validated		iBobbly		[76]	
	Behavior: Strengths and Difficulties Questionnaire	Self-reported and validated		BlueIce		[54,55]	
	Suicide risk: Center for Epidemiologic Studies Depression Scale	Self-reported and validated		myPlan		[59]	
	Suicidal ideation: Depressive Symptom Inventory–Suicidality Subscale	Self-reported and validated		iBobbly		[74,76]	

### App Engagement

Four studies reported data on the use of 4 apps: 2 on dating and sexual violence prevention (Co6 [63] and uSafeUS [93]), 1 on bullying and school violence prevention (uSafeHS) [60], and 1 on self-harm or suicide prevention (iBobbly) [74]. Multimedia Appendices 4 and 5 [54,55,57,59-66,70,74,76,79,92,93] provide details on the characteristics and the findings of the included evaluation studies. On the basis of app download and use, the findings of 2 studies indicated that there is a high level of app use among high school students (uSafeUS) [70] and Aboriginal and Torres Strait Islander youth (iBobbly) [74]. Two studies involving college students found generally low app engagement [63,93]. The findings indicated that younger app users had higher engagement levels than older users, and women were more engaged than men [93]. The reasons for low app use among college students included the perceived redundancy with existing smartphone features, college women’s discomfort with group messaging (Co6) [63], and the opinion among older college students that the app was not relevant to their needs (uSafeUS) [61].

### App Users’ Experience

Data on users’ experience of the apps were obtained from 13 studies, with all 4 app categories being covered (Tables 3 and 4; Multimedia Appendices 4 and 5) [54,57, 59-63,65,66,70,74,79,93]. In general, participants liked the app features and perceived the apps to be easy to use and effective. One dating and sexual violence prevention app (myPlan), 1 self-harm and suicide prevention app (iBobbly), and 1 bullying and school violence prevention app (+FORT) were perceived to be judgment free by youth [60,66,74]. Among the 9 apps, the features perceived to be the most useful included map features, personalized action plan, mood diary, and bullying prevention strategies. The appealing features, confidentiality, accessibility, ease of use, and useful resources were common facilitators of app use among youth. In addition, the judgment-free nature of some apps facilitated their use. By contrast, the repetitive, redundant nature and lack of specific resource information were among the reported barriers to app use.

**Table 3:** Youth’s perceptions of mobile personal safety apps.

Users’ perception and descriptors		Homeless youth support	Dating and sexual violence prevention					Bullying and school violence prevention			Self-harm and suicide prevention	
		YTH StreetConnect [62]	Circle of 6 [63]	Liad@s [57]	myPlan [59,65,66,79]	USafeUS [61,93]	+FORT [60]		uSafeHS [70]	BlueIce [54,55]		iBobbly [74]
**Engagement**												
	Accessible						✓			✓		✓
	Easy to use	✓	✓		✓	✓			✓	✓		
	Fun or enjoyable	✓										
	Favorable features	✓	✓		✓	✓			✓	✓		
**Aesthetics**												
	Appealing design	✓			✓							
**Safety, privacy, and trust**												
	Private or confidential									✓		✓

**Table 4:** Youth’s perceptions of the usefulness of mobile personal safety apps.

Users’ perception and descriptors		Homeless youth support	Dating and sexual violence prevention					Bullying and school violence prevention			Self-harm and suicide prevention	
		YTH StreetConnect [62]	Circle of 6 [63]	Liad@s [57]	myPlan [59,65,66,79]	USafeUS [61,93]	+FORT [60]		uSafeHS [70]	BlueIce [54,55]		iBobbly [74]
**Subjective quality**												
	Useful	✓	—^a^	—	✓	—	✓		—	—		✓
	Effective	✓	Mixed	✓	✓	✓	✓		✓	✓		✓
	Judgment free or shame free	—	—	—	✓	—	✓		—	—		✓
	Would use again	✓	—	—	—	—	—		✓	✓		—
	Worth recommending	—	Mixed	—	—	—	—		—	✓		✓
	Most useful or helpful features	Map feature (Resource Finder)	—	—	Personalized action plan and Myth section	—	Bullying prevention strategies and journal		—	Mood diary, mood lifter, and emergency numbers		—
	Preferred features	—	Location feature	—	Danger assessment tool	Customizable and educational and off-campus resources	—		Customizable	Mood diary		—
	Nonpreferred features	Lack of specific resource information	Redundant or unnecessary	Not helpful	Lack of information on emotional abuse	Not helpful	—		—	Repetitive and not challenging		—

^a^No or not reported.

Both the youth and service providers who participated in evaluating the homeless youth support app, YTH StreetConnect [62], enjoyed using the app and considered it to be accessible, appropriate, and confidential for locating services. None of the evaluation studies analyzed the privacy or confidentiality of the apps.

For the dating and sexual violence prevention apps, the views of youth and other stakeholders, including college campus administrators and crisis center advocates, were captured [59,61,63,65,66,79,93]. Participants generally had positive perceptions of the apps’ sexual violence–related resources and supporting features. For example, Lindsay et al [66] found that women survivors of dating violence who attended college found myPlan to be “useful, innovative, and effective” in conveying information regarding dating violence and relationship safety. In particular, the “My Plan” (personalized safety plan) feature was found to be the most useful feature [66]. By contrast, Debnam and Kumodzi [79] found that among participants who represented a sexually diverse group of adolescents, there was an intolerance to gender-conforming language in the app, which targeted females only. They strongly believed that men can be victims too, and that conversely, women can also be perpetrators. Consequently, participants recommended that the app be modified to reflect a more inclusive group to users with diverse sexual orientations and to have a greater focus on safety dilemmas faced by youth, such as emotional or psychological abuse and power imbalance induced by age difference. Because of that research, a lesbian, gay, bisexual, transgender, queer (LGBTQ) version of the myPlan app was developed and later released [79]. In addition, Potter et al [61] found that while most college students and other stakeholders who used uSafeUS agreed on the need of mobile apps to protect against sexual violence, most of the graduate students (particularly older students) as well as commuter students indicated that they did not perceive the app as being applicable or relevant to their needs.

Across the 2 studies that evaluated bullying and school violence prevention apps among high school students, participants found the app features to be favorable and felt that the apps were useful personal safety tools [60,70]. For example, all the survivors of bullying who used +FORT felt that it may be a beneficial tool, with 1 participant stating, “We talked about it [bullying] during three hours at school and I learned more about it with Stronger than Bullying (as the app was initially called) in five minutes” [60]. uSafeHS users felt that the app could serve as a useful tool for high school students, with all participants who completed the gamified social emotional learning educational modules expressing that their knowledge had improved [70].

Both self-harm and suicide prevention apps were found to be acceptable and helpful to users [54,55,74,76]. In particular, iBobbly was considered culturally appropriate by Aboriginal and Torres Strait Islander youth [74,76]. Although 2 users of BlueIce reported initial concerns that seeing their mood diary full of negative days might not help them, they felt that overall, self-monitoring was beneficial [55].

### Evidence by Outcome

The effect of app use on substance use; decisional conflict; safety behaviors; protective behavior; sexism; ambivalence toward men; love myths; sexual violence; bullying victimization; and mental health issues, such as depression, anxiety, self-harm, and suicide risk, were evaluated in 6 studies (8 articles). All the studies that evaluated effectiveness reported positive outcomes associated with app use in at least 1 outcome measure.

### Substance Use

Two studies reported on substance use, but only 1 assessed the impact of the app on this outcome. A 12-month RCT study involving college women found that there was a reduction in the frequency of alcohol use, getting drunk, binge drinking, and nonmarijuana drug use over time in both the intervention and control groups. However, only the reduction in drunkenness frequency achieved statistical significance (*P=*.001), but there was no significant difference between both groups. Interestingly, there was a slight increase in marijuana use in both groups over time [59]. Although the feasibility and acceptability of Co6 among college women who drink alcohol were assessed, its effects on alcohol and the risk of sexual violence were not assessed. Rather, how app users perceived the app as a sexual violence risk reduction tool was assessed [63].

### Decisional Conflict and Safety Behaviors

One study that evaluated decisional conflict and safety behaviors in college women reported statistically significantly greater improvement in preparedness to make better safety decisions in the intervention group compared to the control group [59]. There were immediate improvements in all decisional conflict subscales in both groups. In particular, participants in the intervention group were statistically significantly better able to weigh the risks and benefits of different safety options compared to those in the control group (*P=*.02). It was found that the number of helpful safety behaviors used on the app increased over time, although there was no statistically significant difference between the intervention and the control groups. There was a statistically significant association between the number of safety behaviors tried and IPV reduction in the intervention group only (*P*<.001) [59].

### Protective Behavior

Four studies that evaluated protective behavior or had themes surrounding that topic found that youth were generally willing to help their friends in risky situations [63,65,79,93]. In 1 study, users of Co6 app expressed almost 3 times more protective behavior in sexually aggressive situations toward friends compared to strangers at 2-month follow-up [63]. Of note, they also expressed greater intention to help friends than strangers at the start of the study. In another study, the myPlan app helped friends of survivors of IPV to understand abusive relationships better and helped them to better understand the severity of violence, identify resource options, and know possible ways to intervene [65]. In evaluating the reasons for downloading uSafeUS, it was found that 90% of college women felt confident that it would provide sufficient resources to help them support a friend who disclosed that they had been sexually assaulted [93]. In another study on the myPlan app, while adolescents expressed willingness to help protect their friends who experience dating violence, they also described the moral distress they experienced regarding protecting themselves over their friends in risky situations [79].

### Sexism, Ambivalence Toward Men, and Love Myths

Sexism was measured in 2 quasi-experimental studies evaluating the Liad@s app. One involved a 2-week intervention involving residents of childcare homes in Spain, who ranged in age from 11 to 18 years [57], and the other involved a 2-hour intervention involving high school students aged 13.9 years, on average [64]. Ambivalence toward men and love myths (distortions about romantic love) were measured only in the study involving residents of childcare homes in Spain [57]. Across the 2 studies, the app was found to be effective in reducing sexism, ambivalence toward men, and love myths. Participants experienced a statistically significant reduction in hostile sexism (*P*=.009) and benevolent sexism post intervention relative to pre intervention (*P*<.001), with greater reductions observed in these variables in the intervention group compared to the control group. The difference was statistically significant only in the study involving high school students [64]. Similarly, there was a significant reduction in ambivalence toward men and distortions of romantic love postintervention relative to pre intervention among residents in a childcare home (*P*=.02 and *P*<.001, respectively) [57]. A statistically significant pre-post difference in distortions of romantic love was observed in the intervention group only. There were no significant gender differences in sexism or myths about romantic love. A statistically significant decrease in hostile sexism with increasing age was observed, and there was also a decrease in paternal resentment with age [57].

### IPV and Other Sexual Violence

One RCT study that evaluated the impact of a personal safety app (myPlan) on IPV among college-going female survivors of IPV between 18 and 24 years of age found that after 12 months of using the app, there was a statistically significant decrease in IPV in both intervention and control groups in all 4 subscales measured (Composite Abuse Scale, traumatic brain injury–related IPV, digital abuse, and reproductive coercion) [59]. The intervention group, however, experienced a statistically significantly greater reduction in reproductive coercion compared to the control group (*P=*.02). In 1 mixed methods study that evaluated sexual victimization among college women, 23% (10/44) of participants who used Co6 reported sexual victimization at 2-month follow-up, which involved unwanted sexual contact (5/10, 50%), completed rape (3/10, 30%), and attempted rape (2/10, 20%) [63]. Most of the perpetrators were friends or acquaintances 44% (4/9), while 33% (3/9) were strangers and 22% (2/9) were their boyfriends. The participants had mixed views on whether the app made them feel safer from sexual violence. App use was low due to perceived redundancy with existing smartphone features and college women’s discomfort with group messaging [63] (Multimedia Appendices 4 and 5).

### School Violence and Bullying Victimization

One study that reported on bullying victimization among high school students found a 2-fold reduction in bullying victimization, which occurred after 4 to 6 weeks of app use (*P*<.001). The reduction in victimization was 16 times greater for the intervention group compared to the control group, who did not receive the app. None of the 5 parents or educators involved in the study believed that the app could jeopardize or conflict with existing services [60]. One study that gathered participants’ input and feedback on the development and testing of a school violence prevention app (uSafeHS) found that the app was well received by youth and appeared to be a useful tool in streamlining all services for homeless or unstably housed youth and their service providers. The impact on safety was, however, not evaluated [70].

### Depression

All 3 studies that reported on depression (2 on self-harm or suicide prevention apps and 1 on a dating and sexual violence prevention app) found a statistically significant reduction among youth who used personal safety apps (*P*<.001 to .02) [55,59,76]. For 1 app (myPlan), there was no difference between the intervention and control arm, whereas for another (iBobbly), the difference between arms was statistically significant. Interestingly, a follow-up study involving participants of the initial iBobbly RCT study found a nonstatistically significant reduction in depression over time [74]. Of note, the sample size of the follow-up study was much smaller than that of the initial study (13 compared to 61).

### Anxiety, Psychological Distress, and Impulsivity

Two studies (3 articles) on self-harm or suicide prevention apps that reported on anxiety-related outcomes found statistically significant reductions in anxiety and psychological distress over time [55,74,76]. One noncontrolled study on BlueIce found a statistically significant reduction in overall scores across all 5 Revised Child Anxiety and Depression Score subscales: panic disorder, separation anxiety disorder, generalized anxiety disorder, social anxiety disorder, and obsessive compulsive disorder (*P*<.001). One RCT on iBobbly reported statistically significant reductions in psychological distress (*P*=.02), which was statistically different from the control (waitlist) arm. However, a follow-up report on iBobbly found nonstatistically significant reductions in psychological distress and impulsivity [74].

### Self-Harm

A pre-post mixed methods study on a self-harm and suicide prevention app found a reduction in self-harming incidents among 33 youth between 12 and 17 years of age who attended mental health services in the United Kingdom [54,55]. The app helped individuals to not act on their urges to self-harm, with 15% (4/26) of those who self-harmed before the study stopping that practice, and a further 58% (15/26) self-harmed less frequently after using the app [55]. A total of 308 incidents of self-harm were prevented during the study, based on historical clinical data and self-reported rates after app use. In total, 27% (7/26) of participants had no reduction in self-harming behavior. No app user felt that the app would increase their thoughts of self-harming, and no adverse events were reported [54].

### Suicide Ideation and Suicide Risk

The 2 studies that evaluated suicide risk–related outcomes among youth reported a reduction after using personal safety apps [59,74]. For 1 dating and sexual violence prevention app, which was used for 12 months, there was a reduction in suicide risk, which was significantly greater in the intervention group relative to the control group (*P*=.048) [59]. For a self-harm or suicide prevention app, which was used for 6 weeks, the reduction in suicide ideation was nonsignificant. In addition, a third study, which evaluated the safety of another self-harm or suicide prevention app, found that no clinician withdrew any participant from the study because of escalated or emergent risk of suicide planning or attempt [55]. The effectiveness of the apps was attributed to several factors, including the provision of distractions and emotional outlets, tracking and recognizing mood patterns, identifying triggers for negative emotions, gaining new perspectives and coping strategies, improving interpersonal communications, and quick access to emergency numbers [54,74].

## Discussion

### Principal Findings

This scoping review identified, categorized, and characterized mobile apps used for personal safety among at-risk children and youth and summarized the findings on app use (engagement), users’ perception, and effectiveness. To the best of our knowledge, this is the first review that provides this type of information on personal safety apps intended to be used autonomously by children and youth. Six (86%) of the 7 studies that evaluated effectiveness reported positive outcomes associated with app use in at least 1 outcome measure, with statistically significant reductions in drunkenness frequency [59], sexism, ambivalence toward men, love myths [57,64], IPV [59], bullying victimization [57], depression [55,59,76], anxiety [54,55], and suicide risk [59] reported over time. In addition, statistically significant differences between intervention and control arms were reported for sexism [57], reproductive coercion (a measure of IPV) [59], and suicide risk [59]. Furthermore, no study reported an increase in harm to participants. These are promising results, which suggest that mobile personal safety apps may be a viable tool for enhancing the safety of children and youth.

From a global personal safety perspective, 100% (13/13) of the included studies were conducted in high-income countries, a reflection of the concentration of mobile app development in such countries. This is highly disproportionate to the high global prevalence and trends in sexual violence, depression, and anxiety in low-income countries [94-96]. A study of global crime patterns during the period 2006 to 2019 revealed that African and Latin American countries experienced the highest levels of various types of crime, followed by Asian countries. Intermediate or relatively low levels of most types of crime were reported for European, North American, and Australian countries [97]. The abovementioned findings demonstrate that more research needs to be undertaken in low- and middle-income countries (LMICs), particularly in areas where mobile phone app use is known to be prevalent. Ding et al [98] had similar findings from their review of mHealth and youth mental health. Furthermore, Madonsela et al [99], in a scoping review on the development and use of mHealth interventions in LMICs, identified only 6 relevant studies from 5 countries. Only 2 of the studies were focused on smartphone apps, and 1 study involved multiple intervention types, including smartphone interventions. Only 1 study that involved a SMS text messaging intervention was focused on self-harm or suicide. The authors concluded that more research is needed to build the evidence base in LMICs to develop this field [99]. Decker et al [100] have made progress in increasing research in LMICs through their RCT to evaluate the efficacy of a culturally and linguistically adapted version of the myPlan app used by women at risk of and experiencing IPV in Nairobi, Kenya. The study, however, had a short duration (3-month follow-up) and was not focused on youth [100].

Considering that only 9 apps met the inclusion criteria, this review suggests that despite the growing number of personal safety apps available on the market, very few are specifically geared toward autonomous use by children and youth. This represents a gap in the mobile personal safety app industry. Many apps are available for use by parents and guardians to track their children, and these have their purpose. However, with a high premium placed on their autonomy, youth generally prefer to use apps that they completely control. As it is for mHealth apps [100], the field of personal safety apps offers both opportunities and risks. For any app, but particularly for personal safety apps geared toward autonomous youth control, it is important that the development be undertaken with target user engagement and for the app to be based on scientific evidence and be well validated. The review found that for most of the apps (8/9, 89%), relevant theories or expertise were used in their development. This indicates that, in general, measures are being taken to ensure that personal safety apps for youth are developed based on sound theories and evidence.

A common feature among the 9 apps is the inclusion of educational content. Interestingly, for 2 apps—1 bullying and school violence prevention app [70] and 1 self-harm and suicide prevention app [74]—youth desired more educational content. In contrast, for the evaluation of 1 dating and sexual violence prevention app, college women found the explanatory video for first-time users “cumbersome” and suggested using dialogue boxes with brief instructions that pop up [61]. The review findings suggest that while younger youth generally have positive perceptions of personal safety apps and highly value them, older youth tend to find them less valuable. As victimization oftentimes begins during adolescence and prevails into adulthood [10], and with the appetite for personal safety apps apparently lessening with age, it may be prudent for more research and development to be focused on the younger segment of the youth demographic [10].

The features perceived to be the most useful included map features (homeless youth support), personalized action plan and myth-debunking sections (dating and sexual violence protection), bullying prevention strategies (bullying and school violence prevention), mood diary, and emergency numbers (self-harm and suicide prevention). The danger assessment tool featured in the myPlan app is unique among the included apps. This user-preferred app feature is a validated tool which provides both numerical and graphical displays of the assessed risk for repeated severe IPV [59,65,66]. Such a feature is potentially very valuable in sexual violence prevention. Common facilitators of app use among youth were the appealing features, confidentiality, accessibility, ease of use, and useful resources. However, the repetitive, redundant nature and lack of specific resource information were barriers to app use. For example, for the YTH StreetConnect app, homeless youth found the lack of specific information such as the number of available beds available at a given shelter to be a barrier to app use [62]. Features that youth suggested for app development or improvement included ambiguous name and branding, an easy delete option, a panic button option, bystander-focused intervention for reducing dating violence, and gamification (Multimedia Appendix 5 [54,55,57,59-66,70,74,76,79,92,93]). As target users’ perception is of utmost importance to app development, these findings are of relevance for app developers.

In 5 (38%) of the 13 included evaluation studies, the period of app engagement did not extend beyond 6 weeks [57,60,63,64,74,76]. In 1 study on users’ experience, app use was as short as 20 to 40 minutes [65]. The longest period reported was 12 months in an RCT study [59]. This reveals that a high proportion of the evaluation studies involved very short app engagement periods, which leaves us to wonder to what extent the results can be related to the real-life context. In particular, no conclusion can be drawn about the long-term effectiveness of personal safety apps for children and youth based on this review. Further studies involving longer app engagement periods and follow-up times need to be undertaken to shed light on effectiveness in the long term.

Regarding measurement outcomes, self-reported measures were used for all but 3 distinct outcomes measured (2 on app use and 1 on self-harming frequency) among all the included studies. While self-reported measures are a valuable tool in public health research, they can inadvertently be affected by various biases, particularly information bias. The use of validated tools, however, can markedly strengthen the validity of self-reported measurement tools. Most of the included studies incorporated the use of validated measures for evaluating effectiveness (6/7, 86% apps). However, only 2 (22%) of the 9 validated measures used were specifically geared toward youth, namely, the Multidimensional Peer-Victimization Scale [60] and the Revised Child Anxiety and Depression Score [54,55]. It may be beneficial for future research to focus on developing and validating tools for evaluating mobile personal safety apps specifically for youth to improve the validity of such research involving youth. In addition, the outcome measurements directly related to victimization or risky behavior were generally based only on a reduction in frequency. Although these measures are useful, the incorporation of measures of the nature or severity of victimization and risky behavior would only serve to improve upon the quality of research conducted.

### Limitations

This review was conducted in accordance with best practices in conducting scoping reviews. However, some limitations exist. Only articles written in English were included; therefore, some relevant articles could possibly have been missed. In addition, a systematic app search was not a part of the search process. Such a search could have potentially identified apps that could have directed the search for evaluation studies. As apps with no available information on users’ experience, app engagement, or impact were outside the inclusion criteria, we believe that a targeted database search along with a search of the gray literature was sufficient and effective in yielding relevant articles. A broad range of study designs and methodologies were included; hence, no quality assessment of included articles was done. However, quality assessment is not a requirement of scoping reviews. The short durations of app use for most of the studies limit the strength of the review findings. In addition, all the evaluation studies used self-reported data, which are more subject to bias than objectively determined measures. The inclusion of validated tools in evaluating all the apps, however, counteracts this limitation to some extent. It is challenging to realistically compare the use, users’ perception, and impact of the apps due to the variability of outcome measures and methodologies. Finally, the generalizability of the findings is limited due to the heterogeneity in participant characteristics across different apps and studies, variations in intervention durations, a small number of apps within each category, and the small sample sizes in several studies.

### Implications for App Development and Future Research

This review sheds light on youth’s experience and perception of personal safety apps geared toward their use. As target users’ perception is of critical importance to app development, these findings are of relevance for app developers. The features perceived to be the most useful included maps, personalized action plans, mood diaries, violence prevention strategies, and emergency numbers. Appealing features, confidentiality, accessibility, ease of use, and useful resources motivated youth to use the apps. Of note, the inclusion of validated danger assessment tools such as the one included in the myPlan app [59,65,66] may be a very useful feature for personal safety apps in general. With the constant evolution of technology, the information included in apps can quickly become obsolete. Therefore, co-design methodologies are essential for increasing and sustaining youth engagement as well as increasing the likelihood of universal acceptability [101]. App developers should, therefore, ensure that youth collaborate in app design and development and that app information is regularly updated, including specific details on helpful resources for at-risk youth. In addition, it may be prudent for more research and development to focus on the younger segment of the youth demographic, given that this group of youth appears to value personal safety apps more compared to older ones [10].

All 9 included apps featured educational content. Inclusion of a test-retest knowledge assessment would be a useful feature to provide basic data on the effectiveness of the apps in increasing knowledge [13] and should be considered in developing these apps. A noteworthy, but not surprising, finding of this review is that youth are generally willing to help their friends in risky situations [63,65,79,93]. This information can be harnessed by app developers. It is potentially very useful to routinely develop companion apps for personal protection apps that target friends of at-risk youth or include features in the apps that allow them to be customized for friends of at-risk youth.

Despite the availability of personal safety apps, the scoping literature review found that there were not many that specifically target youth. Furthermore, all the included studies were conducted in high-income countries, and a high proportion of the evaluation studies had short durations. Most of the evaluation measures were self-reported, and the validated tools were generally not specifically geared toward youth. In addition, some of the outcome measurements were based only on a reduction in frequency. Importantly, the review found that the interest in personal safety apps appears to diminish with age. In light of the abovementioned findings, to better serve the global youth population and to more robustly determine effectiveness, including over the long term, future studies should be conducted as follows: (1) increase focus on development of apps that target youth, particularly the younger demographic; (2) conduct longitudinal studies to determine long-term effectiveness; (3) conduct studies in LMICs; (4) incorporate objective outcome measures into studies; (5) develop standardized measures for evaluating the effectiveness of apps specifically geared toward youth; and (6) include outcome measures that are focused on extent or severity (in addition to frequency of occurrence).

### Implications for Policy Making

Smartphone personal safety apps cannot be considered the panacea for violence against children and youth. Nonetheless, the limited studies available suggest that if personal safety apps are designed based on strong evidence, integrated appropriately into existing interventions, and used effectively, they have the potential to serve as valuable tools for personal safety and, by extension, global health. As is true for personal safety apps for the older demographic, few studies have associated evidence on effectiveness, and among those that do, numerous limitations reduce their generalizability. In addition, there was no focus on privacy and confidentiality in the included evaluation studies. Strong evidence on the effectiveness and security of personal safety apps is needed for them to be fit for integration into interventions used in school, clinical, police, community services, and other settings. As has been recommended for mHealth apps, stringent standards for providing personal safety apps should be established and incorporated into the submission processes used by app stores. In addition, it is imperative that experts in the various fields (education, health care, social security, etc) play a more central role in developing, recommending, and distributing these apps. Furthermore, systematic frameworks to facilitate the translation of personal safety apps into schools, clinical settings, etc would be required.

### Conclusions

The results of this scoping review indicate that mobile personal safety apps generally seem to be effective in reducing harm to at-risk children and youth, with no associated adverse events. Although the findings are promising, several factors limit the robustness of the evidence. Recommendations for future research to improve upon the current state of evidence and availability of effective personal safety apps for children and youth have been made, such as the development of apps that specifically target youth, undertaking studies in LMICs, conducting longitudinal studies, and incorporating objective outcome measures into studies such as the number and nature of reports of victimization to authorities and pre-post professional psychological assessments of risk for self-harm or suicide. Recommendations for app development include incorporating features such as maps, personalized action plans, mood diaries, violence prevention strategies, test-retest knowledge assessments, and validated danger assessment tools. Another recommendation is for app developers to develop companion personal safety apps that target friends of at-risk youth or include features that allow them to be customized for friends’ use, in light of the willingness of youth to help their friends in risky situations. Strong evidence on the effectiveness and security of personal safety apps is needed for them to be fit for integration into interventions used in school, clinical, police, community services, and other settings. There is yet a far way to go in that regard.

## Appendix

Multimedia Appendix 1 PRISMA-ScR (Preferred Reporting Items for Systematic Reviews and Meta-Analyses extension for Scoping Reviews) checklist.

Multimedia Appendix 2 The framework by Jeminiwa et al [51] modified for evaluating the quality of mobile personal safety apps for youth.

Multimedia Appendix 3 Characteristics of the mobile personal safety apps for youth.

Multimedia Appendix 4 Characteristics of the studies evaluating mobile personal safety apps for youth.

Multimedia Appendix 5 Findings of the studies evaluating mobile personal safety apps for youth.

## Abbreviations

Co6: Circle of 6
IPV: intimate partner violence
LGBTQ: lesbian, gay, bisexual, transgender, queer
LMIC: low- and middle-income country
mHealth: mobile health
PRISMA: Preferred Reporting Items for Systematic Reviews and Meta-Analyses
PRISMA-ScR: Preferred Reporting Items for Systematic Reviews and Meta-Analyses Extension for Scoping Reviews
RCT: randomized controlled trial

## References

[1] Mercy JA, Hillis SD, Butchart A, Bellis MA, Ward CL, Fang X, Rosenberg ML, Mock CN, Nugent R, Kobusingye O, Smith KR (2017). Interpersonal violence: global impact and paths to prevention. Injury Prevention and Environmental Health. Third Edition.

[2] Xue J, Hu R, Zhang W, Zhao Y, Zhang B, Liu N, Li SC, Logan J (2021). Virtual reality or augmented reality as a tool for studying bystander behaviors in interpersonal violence: scoping review. J Med Internet Res.

[3] Interpersonal violence prevention. Texas A and M University.

[4] Maxwell L, Sanders A, Skues J, Wise L (2020). A content analysis of personal safety apps: are they keeping us safe or making us more vulnerable?. Violence Against Women.

[5] Ford K, Bellis MA, Judd N, Griffith N, Hughes K (2022). The use of mobile phone applications to enhance personal safety from interpersonal violence - an overview of available smartphone applications in the United Kingdom. BMC Public Health.

[6] Gabor T (2015). Costs of crime and criminal justice responses. Public Safety Canada.

[7] An estimation of the economic impact of violent victimization in Canada, 2009. Government of Canada.

[8] Waters HR, Hyder AA, Rajkotia Y, Basu S, Butchart A (2005). The costs of interpersonal violence--an international review. Health Policy.

[9] Senior M, Fazel S, Tsiachristas A (2020). The economic impact of violence perpetration in severe mental illness: a retrospective, prevalence-based analysis in England and Wales. The Lancet Public Health.

[10] Basile KC, Clayton HB, DeGue S, Gilford JW, Vagi KJ, Suarez NA, Zwald ML, Lowry R (2020). Interpersonal violence victimization among high school students - youth risk behavior survey, United States, 2019. MMWR Suppl.

[11] Midei AJ, Matthews KA (2011). Interpersonal violence in childhood as a risk factor for obesity: a systematic review of the literature and proposed pathways. Obesity Rev.

[12] (2020). Global status report on preventing violence against children 2020. World Health Organization.

[13] Draughon Moret J, Todd A, Rose L, Pollitt E, Anderson J (2022). Mobile phone apps for intimate partner and sexual violence prevention and response: systematic search on app stores. JMIR Form Res.

[14] Whitfield CL, Anda RF, Dube SR, Felitti VJ (2003). Violent childhood experiences and the risk of intimate partner violence in adults: assessment in a large health maintenance organization. J Interpers Violence.

[15] Phoenix J, Francis BJ (2022). Police risk assessment and case outcomes in missing person investigations. Police J.

[16] Missing persons. Edmonton Police Service.

[17] Armitage R (2021). Bullying in children: impact on child health. BMJ Paediatrics Open.

[18] Glew G, Rivara F, Feudtner C (2000). Bullying: children hurting children. Pediatr Rev.

[19] (2024). Adolescent and young adult health. World Health Organization.

[20] Adolescent health in the South-East Asia region. World Health Organization.

[21] Coming of age: adolescent health. World Health Organization.

[22] Brignone L, Edleson JL (2019). The dating and domestic violence app rubric: synthesizing clinical best practices and digital health app standards for relationship violence prevention smartphone apps. Int J Hum Comput Interact.

[23] Number of smartphone users worldwide from 2014 to 2029. Statista.

[24] Number of smartphone mobile network subscriptions worldwide from 2016 to 2023, with forecasts from 2023 to 2028. Statista.

[25] Aguirre RT, McCoy MK, Roan M (2013). Development guidelines from a study of suicide prevention mobile applications (apps). J Technol Human Serv.

[26] Zilka GC (2018). Аlways with them: smartphone use by children, adolescents, and young adults—characteristics, habits of use, sharing, and satisfaction of needs. Univ Access Inf Soc.

[27] (2024). How many people own smartphones? (2024-2029). What's the Big Data.

[28] (2024). Mobile internet usage in Canada. Statista.

[29] (2022). At what age do kids start getting smartphones?. Marketing Charts.

[30] Anderson K, Burford O, Emmerton L (2016). Mobile health apps to facilitate self-care: a qualitative study of user experiences. PLoS One.

[31] Peng W, Kanthawala S, Yuan S, Hussain SA (2016). A qualitative study of user perceptions of mobile health apps. BMC Public Health.

[32] Sama PR, Eapen ZJ, Weinfurt KP, Shah BR, Schulman KA (2014). An evaluation of mobile health application tools. JMIR Mhealth Uhealth.

[33] Vaghefi I, Tulu B (2019). The continued use of mobile health apps: insights from a longitudinal study. JMIR Mhealth Uhealth.

[34] Muntaner-Mas A, Martinez-Nicolas A, Lavie CJ, Blair SN, Ross R, Arena R, Ortega FB (2019). A systematic review of fitness apps and their potential clinical and sports utility for objective and remote assessment of cardiorespiratory fitness. Sports Med.

[35] Park M, Yoo H, Kim J, Lee J (2018). Why do young people use fitness apps? Cognitive characteristics and app quality. Electron Commer Res.

[36] West JH, Hall PC, Hanson CL, Barnes MD, Giraud-Carrier C, Barrett J (2012). There's an app for that: content analysis of paid health and fitness apps. J Med Internet Res.

[37] Wells C, Spry C (2022). An Overview of Smartphone Apps: CADTH Horizon Scan.

[38] Kai OJ (2019). Developing a real time digital emergency personal safety app to assist in emergency situations using best time. Universiti Tunku Abdul Rahman.

[39] Doria N, Ausman C, Wilson S, Consalvo A, Sinno J, Boulos L, Numer M (2021). Women’s experiences of safety apps for sexualized violence: a narrative scoping review. BMC Public Health.

[40] Kanagaraj SA, Arjun G, Shahina A (2013). Cheeka: a mobile application for personal safety. Proceedings of the IEEE International Conference on Collaborative Computing: Networking, Applications and Worksharing.

[41] McGrath S (2016). Mobile apps can enhance personal safety efforts. Stud Aff Today.

[42] Oksiutycz A, Lubinga E (2021). Factors affecting the adoption of personal safety apps among millennials in Johannesburg, South Africa. S Afr J Inf Manag.

[43] O'Campo P, Velonis A, Buhariwala P, Kamalanathan J, Hassan MA, Metheny N (2021). Design and development of a suite of intimate partner violence screening and safety planning web apps: user-centered approach. J Med Internet Res.

[44] Sumra M, Asghar S, Khan KS, Fernández-Luna JM, Huete JF, Bueno-Cavanillas A (2023). Smartphone apps for domestic violence prevention: a systematic review. Int J Environ Res Public Health.

[45] Eisenhut K, Sauerborn E, García-Moreno C, Wild V (2020). Mobile applications addressing violence against women: a systematic review. BMJ Glob Health.

[46] Sinha S, Shrivastava A, Paradis C (2020). A survey of the mobile phone-based interventions for violence prevention among women. Adv Soc Work.

[47] Arksey H, O'Malley L (2005). Scoping studies: towards a methodological framework. Int J Soc Res Methodol.

[48] Mak S, Thomas A (2022). Steps for conducting a scoping review. J Grad Med Educ.

[49] Tricco AC, Lillie E, Zarin W, O'Brien KK, Colquhoun H, Levac D, Moher D, Peters MD, Horsley T, Weeks L, Hempel S, Akl EA, Chang C, McGowan J, Stewart L, Hartling L, Aldcroft A, Wilson MG, Garritty C, Lewin S, Godfrey CM, Macdonald MT, Langlois EV, Soares-Weiser K, Moriarty J, Clifford T, Tunçalp Ö, Straus SE (2018). PRISMA extension for scoping reviews (PRISMA-ScR): checklist and explanation. Ann Intern Med.

[50] Etzion D, Romi S (2015). Typology of youth at risk. Child Youth Serv Rev.

[51] Jeminiwa RN, Hohmann NS, Fox BI (2019). Developing a theoretical framework for evaluating the quality of mHealth apps for adolescent users: a systematic review. J Pediatr Pharmacol Ther.

[52] Stoyanov SR, Hides L, Kavanagh DJ, Zelenko O, Tjondronegoro D, Mani M (2015). Mobile app rating scale: a new tool for assessing the quality of health mobile apps. JMIR Mhealth Uhealth.

[53] Terhorst Y, Philippi P, Sander LB, Schultchen D, Paganini S, Bardus M, Santo K, Knitza J, Machado GC, Schoeppe S, Bauereiß N, Portenhauser A, Domhardt M, Walter B, Krusche M, Baumeister H, Messner EM (2020). Validation of the Mobile Application Rating Scale (MARS). PLoS One.

[54] Grist R, Porter J, Stallard P (2018). Acceptability, use, and safety of a mobile phone app (BlueIce) for young people who self-harm: qualitative study of service users' experience. JMIR Ment Health.

[55] Stallard P, Porter J, Grist R (2018). A smartphone app (BlueIce) for young people who self-harm: open phase 1 pre-post trial. JMIR Mhealth Uhealth.

[56] Wood MA, Ross S, Johns D (2021). Primary crime prevention apps: a typology and scoping review. Trauma Violence Abuse.

[57] Navarro-Pérez JJ, Oliver A, Carbonell Á, Schneider BH (2020). Effectiveness of a mobile app intervention to prevent dating violence in residential child care. Psychosoc Interv.

[58] (2017). Liad@s of the University allows for reduce between 6% and 25% the sexism in teenagers. Universitat de València.

[59] Glass NE, Clough A, Messing JT, Bloom T, Brown ML, Eden KB, Campbell JC, Gielen A, Laughon K, Grace KT, Turner RM, Alvarez C, Case J, Barnes-Hoyt J, Alhusen J, Hanson GC, Perrin NA (2022). Longitudinal impact of the myPlan app on health and safety among college women experiencing partner violence. J Interpers Violence.

[60] Ouellet-Morin I, Robitaille MP, Campbell M, Bauman S (2018). Stronger than bullying, a mobile application for victims of bullying: development and initial steps toward validation. Reducing Cyberbullying in Schools: International Evidence-Based Best Practices.

[61] Potter SJ, Moschella EA, Demers JM, Lynch M (2021). Using mobile technology to enhance college sexual violence response, prevention, and risk reduction efforts. J Technol Human Serv.

[62] Sheoran B, Silva CL, Lykens JE, Gamedze L, Williams S, Ford JV, Habel MA (2016). YTH StreetConnect: development and usability of a mobile app for homeless and unstably housed youth. JMIR Mhealth Uhealth.

[63] Blayney JA, Jenzer T, Read JP, Livingston JA, Testa M (2018). Enlisting friends to reduce sexual victimization risk: there's an app for that… but nobody uses it. J Am Coll Health.

[64] Navarro-Pérez JJ, Carbonell Á, Oliver A (2019). The effectiveness of a psycho-educational app to reduce sexist attitudes in adolescents. Revista de Psicodidáctica (English ed.).

[65] Alhusen J, Bloom T, Clough A, Glass N (2015). Development of the MyPlan safety decision app with friends of college women in abusive dating relationships. J Technol Hum Serv.

[66] Lindsay M, Messing JT, Thaller J, Baldwin A, Clough A, Bloom T, Eden KB, Glass N (2013). Survivor feedback on a safety decision aid smartphone application for college-age women in abusive relationships. J Technol Hum Serv.

[67] Empowering decisions for a safe path forward. myPlan.

[68] uSafeUS mobile app homepage. uSafeUS Mobile App.

[69] +Fort: stronger than bullying. Google Play.

[70] Potter SJ, Moschella-Smith EA, Lynch M (2022). Building a high school violence prevention app to educate and protect students. J Res Technol Educ.

[71] uSafeHS.

[72] BlueIce app. Oxford Health NHS Foundation Trust.

[73] Stallard P, Porter J, Grist R (2016). Safety, acceptability, and use of a smartphone app, BlueIce, for young people who self-harm: protocol for an open phase I trial. JMIR Res Protoc.

[74] Tighe J, Shand F, McKay K, Mcalister TJ, Mackinnon A, Christensen H (2020). Usage and acceptability of the iBobbly app: pilot trial for suicide prevention in aboriginal and Torres Strait islander youth. JMIR Ment Health.

[75] Digital tools and apps. Black Dog Institute.

[76] Tighe J, Shand F, Ridani R, Mackinnon A, De La Mata N, Christensen H (2017). Ibobbly mobile health intervention for suicide prevention in Australian indigenous youth: a pilot randomised controlled trial. BMJ Open.

[77] Tighe J, Shand F, Christensen H The iBobbly app suicide prevention Kimberley trial 2013 – 2015 community report. Black Dog Institute.

[78] Povey J, Mills PP, Dingwall KM, Lowell A, Singer J, Rotumah D, Bennett-Levy J, Nagel T (2016). Acceptability of mental health apps for Aboriginal and Torres Strait Islander Australians: a qualitative study. J Med Internet Res.

[79] Debnam KJ, Kumodzi T (2019). Adolescent perceptions of an interactive mobile application to respond to teen dating violence. J Interpers Violence.

[80] Henry A (2012). Circle of 6 for iPhone and Android prevents violence, gives you a way out of dangerous situations. Lifehacker.

[81] +Fort: stronger than bullying. Axel.

[82] Greenhalgh I, Tingley J, Taylor G, Medina-Lara A, Rhodes S, Stallard P (2021). Beating Adolescent Self-Harm (BASH): a randomised controlled trial comparing usual care versus usual care plus a smartphone self-harm prevention app (BlueIce) in young adolescents aged 12–17 who self-harm: study protocol. BMJ Open.

[83] (2019). Smartphone app BlueIce could help reduce self-harm. The National.

[84] Tingley J, Greenhalgh I, Stallard P (2020). Technology matters: BlueIce – using a smartphone app to beat adolescent self‐harm. Child Adolesc Ment Health.

[85] ‘Prescribed’ smartphone app offers hope to young people who self-harm. University of Bath.

[86] iBobbly: a social and emotional wellbeing app for aboriginal and Torres Strait Islander peoples. Black Dog Institute.

[87] Shand FL, Ridani R, Tighe J, Christensen H (2013). The effectiveness of a suicide prevention app for indigenous Australian youths: study protocol for a randomized controlled trial. Trials.

[88] Circle of 6. Devpost.

[89] Past project: circle of 6. Youth Tech Health.

[90] Liad@s. Google Play.

[91] +Fort: stronger than bullying. Google Play.

[92] iBobbly [Discontinued]. e-Mental Health in Practice.

[93] Potter SJ, Moschella EA, Smith D, Draper N (2020). Exploring the usage of a violence prevention and response app among community college students. Health Educ Behav.

[94] Abrahams N, Devries K, Watts C, Pallitto C, Petzold M, Shamu S, García-Moreno C (2014). Worldwide prevalence of non-partner sexual violence: a systematic review. The Lancet.

[95] Borumandnia N, Khadembashi N, Tabatabaei M, Alavi Majd H (2020). The prevalence rate of sexual violence worldwide: a trend analysis. BMC Public Health.

[96] COVID-19 Mental Disorders Collaborators (2021). Global prevalence and burden of depressive and anxiety disorders in 204 countries and territories in 2020 due to the COVID-19 pandemic. Lancet.

[97] van Dijk J, Nieuwbeerta P, Joudo Larsen J (2021). Global crime patterns: an analysis of survey data from 166 countries around the world, 2006–2019. J Quant Criminol.

[98] Ding X, Wuerth K, Sakakibara B, Schmidt J, Parde N, Holsti L, Barbic S (2023). Understanding mobile health and youth mental health: scoping review. JMIR Mhealth Uhealth.

[99] Madonsela S, Ware LJ, Scott M, Watermeyer J (2023). The development and use of adolescent mobile mental health (m-mhealth) interventions in low- and middle-income countries: a scoping review. S Afr J Psychol.

[100] Decker MR, Wood SN, Hameeduddin Z, Kennedy SR, Perrin N, Tallam C, Akumu I, Wanjiru I, Asira B, Frankel A, Omondi B, Case J, Clough A, Otieno R, Mwiti M, Glass N (2020). Safety decision-making and planning mobile app for intimate partner violence prevention and response: randomised controlled trial in Kenya. BMJ Glob Health.

[101] Malloy JA, Partridge SR, Kemper JA, Braakhuis A, Roy R (2022). Co-design of digital health interventions for young adults: protocol for a scoping review. JMIR Res Protoc.

