# Supplementary material for: Mobile Apps for the Personal Safety of At-Risk Children and Youth: Scoping Review
Source: JMIR Mhealth Uhealth. 2024 Nov 5;12:e58127. doi: 10.2196/58127 (PMC11576608; doi:10.2196/58127)
Supplement: Multimedia Appendix 2 [file mhealth_v12i1e58127_app2.docx]

## **Multimedia Appendix 2.** Dimensions of Jeminiwa’s framework, modified for describing features and evaluating the quality of personal safety apps for youth [1]^a^

| Technical Quality | Engagement | Aesthetics^b^ | Support System | Personal Safety | Autonomy | Safety, Privacy, and Trust | Subjective Quality^b^ |
| --- | --- | --- | --- | --- | --- | --- | --- |
| Efficiency | Interactivity | Layout | Behavior change support | Panic button | Degree of adolescent control | Safety from social stigma | Usefulness |
| Task-technology fit | Customizability | Graphics | Decision support | Self-tracking | Cost | Credibility | Effective-ness |
| Ease of use | Youth-preferred entertainment | Visual appeal | Social support | Fake call/ text | Platforms | Information security | Re-usability |
| Intuitiveness | Gamification |  | Learning support | Emergency call/text |  |  | Recommen-dability |
| Conformity with user expectation | Simplicity of text and data display |  | Institutional support | Incident reporting |  |  |  |
| Error tolerance |  |  |  |  |  |  |  |
| Controllability |  |  |  |  |  |  |  |
| Suitability for learning |  |  |  |  |  |  |  |

^a^The framework was modified by adding the categories: “Aesthetics”, “Personal Safety”, and “Subjective Quality”. Aesthetics was a sub-category of “Engagement” in the original framework. “Condition-specific support” was removed as a “Support System” sub-category.

^b^Addition of “Aesthetics” and “Subjective Quality” categories was guided by guided by Stoyanov et al. [2]

**References**

1. Jeminiwa RN, Hohmann NS, Fox BI. Developing a theoretical framework for evaluating the quality of mHealth apps for adolescent users: a systematic review. J of Pediatr Pharmacol Ther. 2019;24(4):254-69. [doi: [10.5863/1551-6776-24.4.254](https://doi.org/10.5863/1551-6776-24.4.254)]

2. Stoyanov SR, Hides L, Kavanagh DJ, Zelenko O, Tjondronegoro D, Mani M. Mobile app rating scale: a new tool for assessing the quality of health mobile apps. JMIR mHealth and uHealth. 2015;3(1):e27. [doi: [10.2196/mhealth.3422](https://doi.org/10.2196/mhealth.3422)]
