# Supplementary material for: Mobile Apps for the Personal Safety of At-Risk Children and Youth: Scoping Review
Source: JMIR Mhealth Uhealth. 2024 Nov 5;12:e58127. doi: 10.2196/58127 (PMC11576608; doi:10.2196/58127)
Supplement: Multimedia Appendix 3 [file mhealth_v12i1e58127_app3.docx]

**Multimedia Appendix 3.** Characteristics of personal safety apps for youth

| App Name  (Country) | Goal/Target users/Type | Operating system/ Year launched | Developer/ Funding | General description | Features |
| --- | --- | --- | --- | --- | --- |
| Homeless Youth Support | | | | | |
| - YTH StreetConnect (USA) - YTH StreetConnect Pro (USA) | *Goal*   - To connect homeless and unstably housed (H/UH) youth to vital resources [1]   *Target users*   - H/UH youth in Santa Clara County, CA, USA   OR   - Service providers who serve H/UH youth [1]   *Type*   - Educational [1, 2] - Decision aid [1, 2] | *Operating system*   - Android and iOS [1]   *Year*  *launched*   - 2016 (Prototype) [1] | *Developer*   - Santa Clara University Frugal Innovation Hub [1] - Students, Santa Clara University Frugal Innovation Hub [1] - Developed with feedback from H/UH youth and service providers [1]   *Funding*   - US Centers for Disease Control and Prevention Small Business and Innovations Research grant [1] | - Prototype app [1] - Different modalities for youth and service providers - YTH StreetConnect for youth - YTH StreetConnect Pro for service providers [1] - Accessible via Wi-Fi [1] - Helps locate services using visual enhancements [1] - Peer rating system [1] - Online forum [1] - Confidential access to sexual health services [1] - Online referral function [1] | - Features common to both platforms - Location-based database [1] - Interactive mapping [1] - Emergency hotlines [1] - Features on YTH StreetConnect   - Ratings and comments submitted by users   - Sexual health information   - Weekly health tips via text messaging [1] - Features on YTH StreetConnect Pro   - Referral function   - Access to best practices   - Medical questionnaire to assess clients’ homelessness vulnerability and sexual risk [1] |
| Dating/Sexual Violence Prevention | | | | | |
| Circle of Six (USA) | - To incorporate personal and community resources into sexual victimization risk reduction [3]   *Target user*   - Persons at risk of sexual victimization [3-5] - 18-24 years old [4]   *Type*   - Self-tracking [2, 3] - Personal safety [2, 3] - Educational [2, 3] | *Operating system*   - Android and iOS [3, 6]   *Year launched*   - 2012 (prototype) [5] | *Developer*   - Developed by the following multi-skilled app developers: - Deb Levine, Director and Founder of Youth Tech Health (formerly known as ISIS), a non-profit organization that uses social and mobile media for sexual and reproductive health - Christine Corbett Moran, CEO and Co-founder of Kliq, a mobile startup company; also a freelance iPhone developer - Nancy Schwartzman, multimedia consultant and activist with focus on sexual violence prevention - Thomas Cabus, motion graphics designer [4]   *Funding*   - NR - Research funded by Blane Director’s Award for the Development of Innovative Research in Addictions, Research Institute on Addictions, SUNY University at Buffalo [3] | - Prototype app [3] - App users can enlist up to 6 trusted contacts as their safety network [3] - Contacts are editable in app [3] - App icons represent specific action, and send preset group texts to circle [3, 4] | - “Come and get me” (includes GPS location)   - Car icon   - Text reads: "Come and get me. I need help getting home safely. My GPS coordinates are..." [3, 4] - “Call me” - Phone icon - Text reads: "Call and pretend you need me. I need an interruption." [3, 4] - “I need some advice” - Chat icon - Text reads: "I'm looking for information, just letting you know." - Feature links user with risk-assessment tools and information about healthy versus abusive relationships [3, 4] - Love is Respect and RAINN hotlines can be called automatically in case of emergency [4] |
| Liad@s (Spain) | *Goal*   - To promote healthy, respectful, and equal romantic relationships between adolescents [7] - To reduce maladaptive beliefs and attitudes associated with dating violence, namely: romantic love myths and hostile and benevolent dimensions of sexism [7, 8]   *Target user*   - Teenagers/ adolescents [7, 8]   AND   - At risk of gender-based violence [7, 8]   *Type*   - Educational [2, 8] - Psycho-educational [9] - Sexism and romantic love myths prevention [7-10] - Gender-based violence prevention [7, 9] - Dating violence prevention [8] | *Operating system*   - Android and iOS [7-9]   *Year launched*   - NR | *Developer*   - Universitat de València (José Javier Navarro and co-workers) [7]   *Funding*   - NR - Intervention funded by a competitive research and development and innovation program [8] | - Free mobile app [8] - Based on two dimensions related to sexist attitudes - Ambivalent sexism theory - Hostile sexism: discrimination of people of one sex - Benevolent sexism: stereotypes men as superior and women as fragile, leading to gender imbalance - Romantic love myths [7, 8] - Available in Spanish only [10] - Interactive game format [7] | - Game consists of a roulette wheel with sections having different colors - Each section presents a distinct test or message - A monitor determines the cell that player will land on [9] - Activities to foster critical, reflective thinking - Activities promote development of prosocial skills and attitudes - Encourages app user to question socially accepted mistaken beliefs - Helps app user identify negative messages [8, 9] - Designed to encourage the game dynamic (e.g., players can double, lose, or save points) [9] |
| myPlan (USA) | *Goal*   - To help people at risk of experiencing Intimate partner abuse [11]   *Target users*   - Intimate Partner Violence (IPV) victims [11]   *Type*   - Personal safety [2] - Decision aid app [12]   - Interactive and personalized component   - Safety planning component [13] - Educational [11, 12, 14] | *Operating system*   - Android and iOS - Windows, macOS, ChromeOS (for web version) [11]   *Year launched*   - NR | *Developer*   - Nancy Glass, Johns Hopkins University School of Nursing [11] - Codeveloped with relationship violence survivors, friends of survivors, and relationship violence advocates and providers [11]   *Funding*   - The One Love Foundation [11] - National Institute of Mental Health Grant [11] - National Institute of Child Health and Human Development Grant [11] - Office for Victims of Crime Grant [11] | - Free mobile app [11] - Developed based on literature on empowerment, internet safety decision aid, and safety planning [14] - App may be accessed either via mobile device or web anonymously, and password can be created [11, 14] - No account set up required; completely anonymous to use [11] - No password restoration system; need to restart/download the app if password is forgotten [11] - Two modes available: 1 for DV survivors, and the other for friends of DV survivors [15] - PIN protected [11] - Acts as a resource tool for advocates and healthcare providers [11] - Answering question on the app will tailor it to better meet the individual’s need [11] - App can be used to aid decision-making [11] - Personalized safety information and resources can facilitate moving forward with best decision [11] - LGBTQ adults version available [13] - “Dummy code” option (neutral screen) hides app contents if app user is forced to enter a PIN [11] - Changeable icon to hide the app on home screen [11] - Personalized [11] | *DV survivor mode*   - “My Relationship” - Educational information on awareness of myths and attributes of healthy relationships [11, 12, 14] - “My Safety” - Self-assessment with immediate feedback on red flags for unhealthy relationships [12, 14] - Danger Assessment or Danger assessment –Revised for women abused by female partners validated risk assessment tool provides numerical and graphical display of assessed risk for repeated severe IPV [12, 14] - “My Priorities” - Interactive visual aid allows app user to make pairwise comparisons of the importance between priorities such as privacy, safety, feelings for partner, children’s well-being (as appropriate), academic success, and social support/status [12, 14] - “My Plan” - Personalized safety plan based on responses in the previous sections [12, 14]   *Friend of DV survivor mode*   - Similar features to those in DV survivor mode, but tailored to friends of survivors - “Her Relationship” - “Her safety” - “My Priorities” - My safety - My friend’s safety - My friend’s privacy - My friendships - Helping my friends - “My Plan” [15] |
| uSafeUS (USA) | *Goal*   - To prevent and respond to violence experienced by college students [16]   *Target user*   - College students [16]   *Type*   - Decision aid app [2, 16, 17] - Personal safety app [2, 17] - Primary prevention tool   - Educational component   - Also contains secondary and tertiary features [17] | *Operating system*   - Android and iOS [16, 17]   *Year launched*   - In 2015 (formerly called as uSafeNH) [16] | *Developer*   - Undergraduate and graduate students and faculty from various disciplines, including sociology, social work, and computing technology [17] - A retired New Hampshire (NH) state trooper [17] - Director of NH Sexual Assault Response Team (SART) [17] - Coordinator of statewide (NH) Violence Against Women Campus Consortium [17] - Crisis center advocates [17] - Campus administrators [17]   *Funding*   - NH Charitable Foundation [17] | - Free mobile app [16] - Previously known as uSafeNH, which was developed to improve institutional response to campus sexual assault [16] - Interactive prevention features [16] - Allow users’ instant access to emergency channels and related resources both on and away from campus [16] - Allow users’ access to preventive resources with regards to sexual assault, dating violence, and stalking [16] | - Primary prevention feature   - “Helpful Answers”   - Educational content on sexual assault, relationship violence, and stalking [16] - Secondary prevention features - “Time to Leave” - Reduces risk of SV perpetration by enabling user to fake a call or text - “Expect Me” - Enables User to temporarily share GPS location with a friend - “Sound the Alarm” - Enables user to trigger a loud alarm and flashing light - “Angel Drink” - Allows user to alert a bar/restaurant employee that he or she requires help [17] - Customizable tertiary prevention features - “Find Help” - Includes contact information of campus resources, crisis centers, hospitals, police etc. - “What next” - Allows user to explore post-assault reporting options - Resources relating to sexual assault, dating violence, and stalking link [17] |
| Bullying/High School Violence Prevention | | | | | |
| +FORT: Stronger than Bullying (Canada) | *Goal*   - To help youth reduce their bullying experiences - To inform youth about bullying in an original, interactive, and dynamic way - To enhance youth’s understanding of their experience - To encourage youth to try different strategies to reduce bullying [18, 19]   *Target user*  Primary   - Adolescents, 12-16 years of age   AND   - Victims of bullying [18]   Secondary   - Teachers and healthcare professionals [18]*Type* - Education app [2] - Bullying prevention [18-20] | *Operating system*   - Android and iOS [19]   *Year launched*   - NR [18] | *Developer*   - Isabelle Ouellet-Morin and coworkers; University of Montréal, Institut universitaire en santé mentale de Montréal (IUSMM), and AXEL, Québec, Canada [19]   *Funding*   - NR | - Free mobile app [18-20] - Available in French and English [18-20] - App structure and content based on a coordinated sequence of 4 mechanisms of actions central to problem-solving - Learn relevant, empirically based information about bullying - Take a more active role in reducing bullying - Clearly define the bullying problem to adopt more effective strategies to respond to it - Brainstorm and evaluate selected solutions [18, 19] | - Strategies - Different strategies to help diminish bullying provided [18, 19] - List of online and telephone resources with additional strategies [18] - Youth prompted to add their own strategies [18, 19] - Youth prompted to try out the strategies and evaluate them based on 4 dimensions of emotions (anger-serenity, sadness-happiness, insecure-confident, anxiety-calmness) and overall perceptions of usefulness [18, 19] - Journal - App prompts users to answer questions about their bullying experiences (every day, every other day, or once per week) [18, 19] - App compiles information in simple graphs, illustrating the forms of bullying youth experiences most frequently, where it occurs, and whether experiences increase or decrease over time [18, 19] - Graphs may be shared with someone youth trusts via email or in person when youth feels ready [18, 19] - Videos - 10 short educational videos (on cyberbullying, homophobia, consequences of bullying, etc.) that feature well-known personalities popular with youth [18, 20] - Some videos show youth personalities taking a stand against bullying [18] - Badges - Incentives for different levels of app engagement [20] |
| uSafeHS (USA) | *Goal*   - To offer students safe, practical approaches for dealing with risky behaviors, provide education on healthy relationship, and make finding help easier for them in school and beyond [21]   *Target users*   - High school students [21]   *Type*   - Educational app [2] - Personal safety app [2] | *Operating system*   - Android and iOS [21]   *Year launched*  2006 [21] | *Developer*   - Prevention Innovations Research Center of University of New Hampshire [21]   *Funding*   - NR | - App corresponds with a school administrator dashboard [22] - App is both an educational and prevention app (i.e., similar degree of focus on both; typical apps focus on either one) [22] - App development based on three theoretical frameworks: - Social self-identification - Social-ecological model - Consolidated Framework for Implementation Research [22] | Mobile app   - “Learn & Earn”:   - Gamified social emotional learning educational modules [21, 22] - “Time to Leave”   - Allows student to send himself/herself a fake call or text to make it easy to leave an uncomfortable situation - “Find Resources”   - School, community, and national resources [21, 22] - “Report a concern”   - A confidential tip line for reporting incidents or concerns to school administrators in real time [21, 22]   Administrator dashboard   - School resources   - Names, contacts and other categories of the school resources [22] - Community Resources   - Local community resources for students’ use [22] - Online Resources   - Online resources for the students [22] - Push notifications   - A way to communicate with students via push messages sent to their phones [22] - Analytics   - Anonymous data about uSafeHS use on campus by day and by time [22] - Communication tools   - Tools for the school’s use [22] - Incident report settings [22] |
| Self-harm/Suicide prevention | | | | | |
| BlueIce (UK) | *Goal*   - To help young people manage their emotions and reduce urges to self-harm [23]   *Target users*   - Young people attending child and adolescent mental health services (CAMHS) across the UK [24]   And   - Engaging in self-harm [23-29]*Type* - Educational app [2] - Self-harm prevention app [23, 25, 27-29] - Self-help app [28, 29] - Mental health app [23, 25-27, 29] | *Operating system*   - Android and iOS [23, 26, 28, 29]   *Year launched*   - NR | *Developer*   - Paul Stallard from Oxford Health NHS (National Health Services) Foundation and Child and Adolescent Mental Health Group, Department for Health, University of Bath, UK [23-29] - Co developed with young people with history of self-harm [25-29]   *Funding*   - NR - Research project funded by: - NHS England - Health Foundation [29] | - Smartphone app [23-29] - Available only by prescription [23, 26] - Available via license to CAMHS [26] - Developed based on cognitive behavior theory (CBT) and dialectic behavior therapy (DBT) - CBT: behavioral activation, thought challenging activities, mood lifting activities - DBT: mindfulness and distress tolerance activities [27] - BlueIce refers to low mood (Blue) and “in case of emergency” (Ice) [30] - Designed to be used along with face-to-face mental health care [23, 27] - PIN (personal identification number) protected - The personal information the user enters into the app is only stored on user’s device, but may be reviewed with mental health clinician during routine visits [23, 27] | - Mood diary - User rates their mood and may also add notes to record reason for their feeling - User saves rating and notes in a calendar which he or she and therapist can review [27, 28] - Personalized mood lifting section - If mood rating is low, app automatically routes user to mood lifting section; user may access this feature directly from main menu - Helps app user to manage urges to self-harm - 8 personalized activities to enhance mood   - “Get active”: physical activities   - “Do something”: “feel-good activities”   - “Chill and relax”: mindfulness and calming exercises   - “Thinking traps”: a place where thoughts can be recorded, saved, and reviewed   - “Good times”: photo library of happy memories   - “Contact Friends”: 3 contacts whom young person may reach out to when feeling low or in danger of self-harming   - “Listen and Relax”: music library of happy music   - “Ride it Out”: DBT techniques including instructions for an ice dive, a self-soothing exercises, and pros and cons balance sheet for self-harming [25-28] - Emergency contact numbers if urge to self-harm has not reduced - Young person asked to rerate mood after using mood-lifting section; if urge to self-harm has not reduced, young person automatically routed to emergency numbers [23, 25-28] |
| iBobbly (Australia) | *Goal*   - To tackle suicide prevention among Aboriginal and Torres Strait Islanders in a culturally appropriate way [31]   *Target*   - Aboriginal and Torres Strait Islanders - AND - ≥ 15 years of age (youth, in particular [31, 32])   AND   - One or more of the following: - Feeling sad or having low mood - Possibly having thoughts of self-harm [33]   *Type*   - Educational app [2] - Social and emotional wellbeing app [34, 35] - Suicide prevention app [31, 36] - Self-help app [32, 34] - Mental health app [31] | *Operating system*   - Android and iOS [31]   *Year launched*   - 2013 (trial launched) [31] | *Developer*   - Black Dog Institute - Codeveloped with Aboriginal and Torres Strait Islander stakeholders in Kimberley region, north Western Australia [31]   *Funding*   - Australian Government Department of Health - NSW Mental Health Commission - Nib Foundation - Mostyn Foundation - Matana Foundation for Young People - Aon - Samsung (6) | - Internet connectivity not required for app use after downloading [31-33] - Developed based on acceptance and commitment therapy   - Activities include alternatives that help app users accept difficult thoughts and feelings [31, 32, 37]   - Activities help app users regulate and increase the awareness of these feelings rather than struggling against them or avoiding them [31, 32, 37] - Activities help individuals move in positive directions through effective behavior change [37] - Personalized dashboard to track progress [31, 32] - All text accompanied by associated gender-matched audio icon [31, 32, 37] - Original imagery created by Aboriginal and Torres Strait Islander artists and graphic designers to represent the main messages and activities of the therapeutic content [31, 32] - Activities must be completed sequentially [32] - A summary page encourages reflection [37] | Main features   - “How do I feel” [35] - 3 self-assessments [31, 32, 36, 37] - Walks user through quick check and gives feedback on how user is tracking [33] - “Stuff I can use” [35] - 2 of 3 content modules aimed at addressing thoughts, emotions, and values - Module 1: app user learns to identify thoughts, feelings, and behaviors, and learn distancing techniques - Module 2: app user learns to regulate their emotions through mindfulness, acceptance, and self-soothing social activities [31-33, 36] - “How I’m gonna beat this” [35] - 1 of 3 content modules aimed at addressing thoughts, emotions, and values [31-33, 36] - Module 3: App user learns to identify his/her values; sets small, achievable goals towards living by these value; and receives a personalized action plan based on their responses [32] - “Help” [35] - Provides help and support options - Emergency contact information for 24-hour helplines [31, 32] |

**References**

1. Sheoran B, Silva CL, Lykens JE, Gamedze L, Williams S, Ford JV, et al. YTH StreetConnect: Development and Usability of a Mobile App for Homeless and Unstably Housed Youth. JMIR mHealth and uHealth. 2016;4(3):e82. [doi: [10.2196/mhealth.5168](https://doi.org/10.2196/mhealth.5168)]

2. Wood MA, Ross S, Johns D. Primary crime prevention apps: a typology and scoping Review. Trauma, Violence, & Abuse. 2022;23(4):1093-110. [doi: [10.1177/1524838020985560](https://doi.org/10.1177/1524838020985560)]

3. Blayney JA, Jenzer T, Read JP, Livingston JA, Testa M. Enlisting friends to reduce sexual victimization risk: There's an app for that… but nobody uses it. J Am Coll Health. 2018;66(8):767-73. [doi: [10.1080/07448481.2018.1446439](https://doi.org/10.1080/07448481.2018.1446439)]

4. Devpost Inc. Circle of 6 2011. URL: <https://devpost.com/software/circle-of-6> [accessed 2024-05-03]

5. Youth Tech Health. Past project: Circle of 6 2024. URL: <https://yth.org/projects/circle-of-6/> [accessed 2023-03-21]

6. Lifehacker. Circle of 6 for iPhone and Android prevents violence, gives you way out of dangerous situations 2012. URL: <https://lifehacker.com/circle-of-6-for-iphone-and-android-prevents-violence-g-5941286> [accessed 2023-08-15]

7. Universitat de València. Liad@s of the University allows for reduce between 6% and 25% the sexism in teenagers 2023. URL: <https://www.uv.es/uvweb/college/en/profile/liad-s-university-allows-reduce-6-25-sexism-teenagers-1285950309813/Novetat.html?id=1286026137921&plantilla=UV/Page/TPGDetaill> [accessed 2023-03-21]

8. Navarro-Pérez JJ, Oliver A, Carbonell Á, Schneider BH. Effectiveness of a mobile app intervention to prevent dating violence in residential child care. Psychosoc Interv. 2020;29(2):59-66. [doi: [10.5093/pi2020a3](https://doi.org/10.5093/pi2020a3)]

9. Navarro-Pérez J-J, Carbonell Á, Oliver A. The effectiveness of a psycho-educational app to reduce sexist attitudes in adolescents. Rev Psicodidáct (English ed.). 2019;24(1):9-16. [doi: [10.1016/j.psicod.2018.07.002](https://doi.org/10.1016/j.psicod.2018.07.002)]

10. ARTEC - Universidad de Valencia. Liad@s 2019. URL: <https://play.google.com/store/apps/details?id=es.uv.artec.Liados&hl=en_GB&pli=1> [accessed 2024-03-05]

11. Myplanapp.org. myPlan: Empowering decisions for a safe path forward 2023. URL: <https://myplanapp.org> [accessed 2023-08-15]

12. Lindsay M, Messing JT, Thaller J, Baldwin A, Clough A, Bloom T, et al. Survivor Feedback on a Safety Decision Aid Smartphone Application for College-Age Women in Abusive Relationships. J Technol Hum Serv. 2013;31(4):368-88. [doi: [10.1080/15228835.2013.861784](https://doi.org/10.1080/15228835.2013.861784)]

13. Debnam K, Kumodzi T. Adolescent Perceptions of an Interactive Mobile Application to Respond to Teen Dating Violence. J Interpers Violence. 2021;36(13-14). [doi: [10.1177/0886260518821455](https://doi.org/10.1177/0886260518821455)]

14. Glass N, Clough A, Messing J, Bloom T, Brown M, Eden K, et al. Longitudinal Impact of the myPlan App on Health and Safety Among College Women Experiencing Partner Violence. J Interpers Violence. 2022;37(13-14). [doi: [10.1177/0886260521991880](https://doi.org/10.1177/0886260521991880)]

15. Alhusen J, Bloom T, Clough A, Glass N. Development of the MyPlan Safety Decision App with Friends of College Women in Abusive Dating Relationships. J Technol Hum Serv. 2015;33(3):263-82. [doi: [10.1080/15228835.2015.1037414](https://doi.org/10.1080/15228835.2015.1037414)]

16. Usafeus.org. uSafeUS: placing sexual assault prevention and response resources into the hands of students and their allies 2022. URL: <https://usafeus.org/> [accessed 2023-08-15]

17. Potter SJ, Moschella EA, Demers JM, Lynch M. Using Mobile Technology to Enhance College Sexual Violence Response, Prevention, and Risk Reduction Efforts. J Technol Hum Serv. 2022;40(1):25-46. [doi: [10.1080/15228835.2021.1929665](https://doi.org/10.1080/15228835.2021.1929665)]

18. Ouellet-Morin I, Robitaille M-P. Stronger than Bullying, a mobile application for victims of bullying: development and initial steps toward validation. In: Campbell M, Bauman S, editors. Reducing cyberbullying in shools: international evidence-based best practices. Elsevier Inc.; Academic Press; 2018. p. 159-74.

19. Centre Axel. +FORT: Stronger than Bullying 2023. URL: <https://centreaxel.com/en/projects/stronger-than-bullying/> [accessed 2023-08-15]

20. Centre de recherche IUSMM. +Fort: Stronger than Bullying 2022. URL: <https://play.google.com/store/apps/details?id=com.sidekick.ciusss.plusfort&hl=en_CA&gl=US&pli=1> [accessed 2023-08-15]

21. Usafeapp.org. uSafeHS: helping students stay safe and build healthier relationships 2023. URL: <https://www.usafeapp.org> [accessed 2023-08-15]

22. Potter SJ, Moschella-Smith EA, Lynch M. Building a high school violence prevention app to educate and protect students. J Res Technol Educ. 2022:1-19. [doi: [10.1080/15391523.2022.2110336](https://doi.org/10.1080/15391523.2022.2110336)]

23. NHS Oxford Health. BlueIce app 2018. URL: <https://www.oxfordhealth.nhs.uk/blueice/> [accessed 2024-05-03]

24. University of Bath. ‘Prescribed’ smartphone app offers hope to young people who self-harm 2023. URL: <https://www.bath.ac.uk/case-studies/prescribed-smartphone-app-offers-hope-to-young-people-who-self-harm/> [accessed 2023-08-15]

25. Tingley J, Greenhalgh I, Stallard P. Technology Matters: BlueIce – using a smartphone app to beat adolescent self‐harm. Child and Adolescent Mental Health. 2020;25(3):192-4. [doi: [10.1111/camh.12397](https://doi.org/10.1111/camh.12397)]

26. Stallard P, Porter J, Grist R. A Smartphone App (BlueIce) for Young People Who Self-Harm: Open Phase 1 Pre-Post Trial. JMIR mHealth and uHealth. 2018;6(1):e32. [doi: [10.2196/mhealth.8917](https://doi.org/10.2196/mhealth.8917)]

27. Stallard P, Porter J, Grist R. Safety, acceptability, and use of a smartphone app, BlueIce, for young people who self-rarm: protocol for an open phase I trial. JMIR Res Protoc. 2016. [doi: [doi:10.2196/resprot.6525](https://doi.org/doi:10.2196/resprot.6525)]

28. Greenhalgh I, Tingley J, Taylor G, Medina-Lara A, Rhodes S, Stallard P. Beating Adolescent Self-Harm (BASH): a randomised controlled trial comparing usual care versus usual care plus a smartphone self-harm prevention app (BlueIce) in young adolescents aged 12–17 who self-harm: study protocol. BMJ Open. 2021;11(11):e049859. [doi: [10.1136/bmjopen-2021-049859](https://doi.org/10.1136/bmjopen-2021-049859)]

29. Grist R, Porter J, Stallard P. Acceptability, Use, and Safety of a Mobile Phone App (BlueIce) for Young People Who Self-Harm: Qualitative Study of Service Users’ Experience. JMIR Mental Health. 2018;5(1):e16. [doi: [10.2196/mental.8779](https://doi.org/10.2196/mental.8779)]

30. National Newsdesk. Smartphone app could help reduce self-harm, research suggests 2019. URL: <https://www.thenational.scot/news/17807482.smartphone-app-blueice-help-reduce-self-harm/> [accessed 2024-03-05]

31. Tighe J, Shand F, Mckay K, Mcalister T-J, Mackinnon A, Christensen H. Usage and Acceptability of the iBobbly App: Pilot Trial for Suicide Prevention in Aboriginal and Torres Strait Islander Youth. JMIR Mental Health. 2020;7(12):e14296. [doi: [10.2196/14296](https://doi.org/10.2196/14296)]

32. Tighe J, Shand F, Ridani R, Mackinnon A, De La Mata N, Christensen H. Ibobbly mobile health intervention for suicide prevention in Australian Indigenous youth: a pilot randomised controlled trial. BMJ Open. 2017;7(1):e013518. [doi: [10.1136/bmjopen-2016-013518](https://doi.org/10.1136/bmjopen-2016-013518)]

33. Tighe J, Shand F, Christensen H. The iBobbly app Suicide Prevention Kimberley Trial 2013 – 2015 Community report. 2017.<https://www.blackdoginstitute.org.au/wp-content/uploads/2020/04/17-08-09_res_ibobblysummarydocument_website_final.pdf?sfvrsn=2>

34. Black Dog Institute. iBobbly app for Aboriginal & Torres Strait Islanders 2023. URL: <https://www.blackdoginstitute.org.au/resources-support/digital-tools-apps/ibobbly/> [accessed 2023-08-21]

35. Black Dog Institute. iBobbly: a social and emotional wellbeing app for Aboriginal and Torres Strait Islander peoples 2020. URL: <https://www.blackdoginstitute.org.au/wp-content/uploads/2020/08/iBobbly-brochure.pdf> [accessed 2024-03-05]

36. Shand FL, Ridani R, Tighe J, Christensen H. The effectiveness of a suicide prevention app for indigenous Australian youths: study protocol for a randomized controlled trial. Trials. 2013;14(1):396. [doi: [10.1186/1745-6215-14-396](https://doi.org/10.1186/1745-6215-14-396)]

37. Povey J, Mills PPJR, Dingwall KM, Lowell A, Singer J, Rotumah D, et al. Acceptability of Mental Health Apps for Aboriginal and Torres Strait Islander Australians: A Qualitative Study. J Med Internet Res. 2016;18(3):e65. [doi: [10.2196/jmir.5314](https://doi.org/10.2196/jmir.5314)]
