# Supplementary material for: Mobile Apps for the Personal Safety of At-Risk Children and Youth: Scoping Review
Source: JMIR Mhealth Uhealth. 2024 Nov 5;12:e58127. doi: 10.2196/58127 (PMC11576608; doi:10.2196/58127)
Supplement: Multimedia Appendix 4 [file mhealth_v12i1e58127_app4.docx]

**Multimedia Appendix 4.** Characteristics of studies evaluating personal safety apps for youth

| First author, year | Aim | Study type and period | Inclusion criteria | Methods | Participants | Outcomes measured |
| --- | --- | --- | --- | --- | --- | --- |
| Homeless Youth Support | | | | | | |
| YTH StreetConnect + YTH StreetConnect Pro (USA) | | | | | | |
| Sheoran et al., 2016 [1] | - To conduct usability and feasibility testing of the app among homeless and unstably housed (H/UH) youth and local technical experts who serve H/UH youth | *Study type*   - Single-arm - Qualitative   *Period*   - *Study*: October 2014 to March 2015 - App use: NR | All of the following:   - Residing in Santa Clara County - 18 – 25 years of age - Reported an H/UH situation   Service providers   - Providing H/UH youth services (including physicians, community center leaders, health providers, and housing directors) | Recruitment and orientation  *H/UH youth*   - Participants were recruited from H/UH services from California Bay Area via flyers posted at clinics, shelters, and on craigslist - One H/UH youth employed to promote the program in shelters - Participants screened via phone for eligibility - Service providers recruited from community - Youths received US $100 gift card for Safeway food retailer - Providers received US $50 gift card   *Data collection*  Phase I   - Formative research and app development   *Providers*  Interviews   - Topics covered: - Phone and tablet usage - Analysis: thematic   Phase II  *Youth*   - Usability testing (via live app) - Topics covered: - Users’ experience - Feasibility - Possible modifications - Analysis: thematic   *Youth*   - Focus groups - Topics covered: - App’s youth friendliness - Accessibility - Usefulness - Analysis: thematic | - H/UH Youth: N = 6 - Providers: NR   Demographics (N = 6)  *H/UH Youth*   - Female: 3 (50%) - Ethnicity (participants checked one or more): - African American: 4 (66.7%) - Hispanic: 3 (50%) - White: 2 (33.3%) - Sexual orientation: - Heterosexual: 5 (83.3%) - Not informed: 1 (16.7%) - Currently homeless or unstably housed: 6 (100%) - Mobile device ownership:   - Owned cellphone: 6 (100%)   - Owned smartphone: 5 (83.3%) | *Youth and service providers*   - Users’ experiences - Feasibility (all by focus group discussions) |
| Dating/Sexual Violence Prevention | | | | | | |
| Circle of 6 (USA) | | | | | | |
| Blayney et al., 2018 [2] | - To assess the feasibility and acceptability of Co6 among college women who drink alcohol - To assess how the app is perceived as a sexual violence risk reduction tool among young women | *Study Type*   - Single-arm - Mixed methods   *Period*   - Study: 2 months (summer/fall 2015) - App use: 2 months (summer/fall 2015) | All of the following:   - Women enrolled in college - 18 – 24 years of age - Own a smart phone - Drank alcohol at least once per week in last 6 months | Recruitment and orientation   - Participants recruited via:   - Newspaper advertisement   - Campus flyers - Participants introduced to Co6 app via brief presentation during baseline session*,* downloaded app, and added up to 6 friends to their circle - Women instructed to use app in drinking contexts such as bars, parties, and dates   Data collection  *Young, college women*  Self-reported questionnaire   - Topics covered: - At baseline: Intention to help - After 2 months app use: - App use - Bystander behavior - Alcohol use - Sexual victimization - Analysis: descriptive statistics   Interview   - 1 hour long - Topics covered: - App use - Impression of app - Feasibility and acceptability of app - Alcohol use - Analysis: descriptive statistics and thematic analysis | - Baseline: N = 68 - 2-month follow-up: N = 44   Demographics (N = 44)   - Mean age (years) 20.11; standard deviation (SD): 1.33 - Female: 100% - Ethnicity:   - White: 23 (52%)   - African American: 7 (16%)   - Asian: 6 (14%)   - Hispanic: 3 (7%)   - Multi-racial/Other: 5 (11%) - Heterosexual: 41 (93%) - College year   - Freshman: 1 (2%)   - Sophomores: 11 (25%)   - Junior: 19 (43%)   - Senior: 13 (30%) - Alcohol use (typical week in past 6 months) - Average reported drinking days: 2.80 days (SD: 1.15) - Average number of drinks consumed: 12.06 (SD: 6.50) - Helping others - “Strong intentions” to help others - Greater intention to help friends than strangers | - Helping others (by 10-item modified intention to help scale and 44-item Bystander Behavior Scale (Banyard’s BystanderScale)) - Feasibility and acceptability of the App (by Co6 app use questionnaire) - App’s value (by app’s utility question, questions on whether app made participants feel safe from SV, and questions regarding whether or not they would recommend app to friend) - Impression of the app (by thematic analysis) - Involvement in risk context: - Sexual victimization (by 11 item-Revised sexual experience survey) |
| Liad@s (Spain) | | | | | | |
| Navarro-Pérez et al., 2020 [3] | - To determine the effectiveness of the Liad@s app in addressing three variables associated with dating violence in adolescents: sexism, romantic love myths, and ambivalence and prejudice towards men - To determine the acceptability of the app | *Study type*   - Quasi-experimental - Quantitative   *Period*   - Study : November 2016 – February 2017 - App use: 2 weeks | All of the following:   - Residing in a child care home in Valencia, Spain - 11 – 18 years of age - Obtain a minimum of 2,500 game points to remain in study (i.e., about 2 hours per week for 2 weeks) | Recruitment and orientation   - Random selection of residential child care homes - Parents or legal guardians provided written consent for participants under 16 years of age - App introduced to participants via face-to-face slide presentation - Participants practiced app in game mode   Data collection   - Intervention: - 90 minutes of physical attendance in homes - App use for 2 months - Control: - No app use during study period - App received 2 weeks after the study period   Questionnaires   - Pre-post controlled study - Before intervention - After intervention (2 weeks) - Topics covered: - Sexism - Ambivalence towards men - Distortions (myths) about romantic love - Analysis: inferential statistics (multivariate analysis of variance)   One-question survey   - Topic covered: acceptability of app - Analysis: descriptive | - N = 71 - Intervention: N = 35 - Control: N = 36   Demographics (N = 71)   - Female: 42.6% - Mean age (years): 14.94; range: 11 – 18 years; SD: 1.39 - Marital status of parents: - Separated or divorced: 56.3% - Parents living together: 31.3% - Single: 12.5% | - Sexism (by Ambivalent Sexism Inventory (ASI) - Ambivalence towards men (by Ambivalence toward Men Inventory, AMI)) - Distortions about romantic love (by Myths, Fallacies and Erroneous Beliefs about the Ideal of Romantic Love Scale) - Acceptability and users’ experiences (by a one-question survey with 3 possible answers: - “the app has not made me change my way of thinking or relating with peers” - “Liad@s have contributed to your appreciation of other points of view” - “Liad@s helped me to mature in this area; I will try to improve my personal relations” |
| Navarro-Pérez et al., 2019 [4] | - To evaluate the effectiveness of Liad@s app in reducing sexist beliefs and attitudes in adolescents | *Study type*   - Quasi-experimental - Quantitative   *Period*   - Study: NR - App use: 4 weeks | - Students at one of four selected high schools in Spain   AND   - Satisfactorily answering 2 screening questions about the app | Recruitment and orientation   - School management teams provided with information about the study - Random selection of schools - Parents or legal guardians provided written consent for participants - 2 female researchers provided information on gender-based violence and introduced to app via 2-hour workshop   Data collection   - Intervention - 2-hour app orientation workshop - App use for 4 weeks - Control - No app use during study period - App received after the study period   Questionnaires   - Pre-post controlled study - Before intervention - After intervention (4 weeks) - Topics covered: - Sexism (hostile and benevolent) - Analysis: inferential statistics (t-tests and multivariate analysis of variance) | - N = 369 - Intervention: N = 196 - Control: N = 173   Demographics   - Intervention   - Female: 104 (53.1%)   - Mean age (in years) (SD): 13.76 (1.19%)   - Middle socioeconomic level: 148 (75.9%)   - Lives with both parents   - Intervention: 133 (67.5%)   - Father employed: 176 (90.7%)   - Mother employed: 147 (75%)   - Uses social networks to socialize: 173 (87.8%)   - Uses social networks everyday: 133 (68.9%) - Control   - Female: 95 (55.9%)   - Mean age (in years) (SD): 13.89 (1.24)   - Middle socioeconomic level: 130 (75.6%)   - Lives with both parents: 126 (72.8%)   - Father employed: 148 (87.1%)   - Mother employed: 132 (76.7%)   - Uses social networks to socialize: 153 (88.4%)   - Uses social networks everyday: 115 (68%) | - Sexism (by Ambivalent Sexism Inventory (ASI)-Adolescents |
| myPlan app (USA) | | | | | | |
| Glass et al., 2022 [5] | - To compare difference in change over time in health and safety outcomes among female college students who used myPlan app compared to those who used usual web based safety planning resources | *Study type*   - Randomized controlled trial (RCT) - Quantitative   *Period*   - Study: July 2015 to October 2017   App use:   - 12 months | All of the following:   - Enrolled in college or university in Oregon or Maryland at least part-time - 18 – 24 years of age - English speaking cisgender and transgender women - Had safe access to email and an internet enabled personal device - Screened positive for intimate partner violence (IPV) | Recruitment and orientation   - Participants recruited from Maryland and Oregon:   - Three four-year public colleges   - One historically Black college   - Two four-year private college   - Three community colleges - Recruitment via online advertisements through:   - Facebook   - Campus bulletins   - Social media   - Student listservs   - Study advertisements shared among students and staff   Data collection   - Participants randomly assigned to intervention and control group - Intervention group received My Plan app - Control group received usual safety planning guided by basic emergency safety planning information provided to students on-campus through IPV websites - Access to a static emergency safety plan with only two resources (a national IPV hotline and national LGBTQ hotline), rather than more comprehensive safety information and resources provided by myPlan - Following randomization, an email with links was sent for introducing and use of the app - US $20 gift card and resource listing of hotlines and websites focused on adolescent relationship abuse given to participants   Survey   - Conducted in-person - Pre-post - Baseline - 6 months - 12 months - Topics covered: - Decisional conflict and decision preparedness - Safety behaviors - Mental health - Depression - Risk for suicide - Analysis: Inferential (mixed effect regression: chi-square and t-tests | - At Baseline: - Control: N = 177 - Intervention: N = 176 - Total: = 353 - At 6 month - Control: N = 173 (98.3% retention) - Intervention: N = 174 (98.3% retention) - Total: N = 347 - At 12 month - Control: N = 171 (96.6% retention) Intervention: N = 175 (98.9% retention) - Total: N = 346   Demographics   - Intervention   - Mean age: 21.23 years (SD: 2.56)   - Race:     - White: 117 (66.48%)     - African American: 38 (21.59%)     - Asian: 15 (8.52%)     - American Indian/Alaska Native/Native Hawaiian/ Pacific Islander: 8 (4.54%)     - Other: 11 (6.25%)   - Ethnicity     - Latina: 22 (12.50%)   - Year in school     - Freshman: 31 (17.61%)     - Sophomore: 44 (25.00%)     - Junior: 44 (25.00%)     - Senior: 49 (27.84%)     - Graduate students: 8 (4.55%)   - Currently live:     - On-campus: 61 (37.65%)     - Off-campus: 101 (62.35%) - Alcohol and drug use - Frequency of alcohol use in last 30 days: 1.82 (SD: 1.46) - Frequency of getting drunk in last 6 months: 2.43 (SD: 1.22) - Frequency of binge drinking in last 2 weeks: 2.01 (SD: 1.24) - Any marijuana use in last 30 days (N): 90 (51.14%) - Any drug use excluding marijuana in last 30 days (N): 43 (25.75%)   - Intimate partner violence - Total Composite Abuse Score (CAS) score: 23.23 (17.00) - Traumatic Brain Injury (TBI) related IPV: 50 (28.41%)   - - Reproductive Coercion (RC): 52 (29.55%)     - Digital Abuse: 3.14 (1.99)   - Depression: 30.98 (19.04)   - Risk for suicide: 79 (45.9%) - Control   - Mean age: 20.61 years (SD: 2.12)   - Race:     - White: 122 (68.16%)     - African American: 31 (17.32%)     - Asian: 25 (13.97%)     - American Indian/Alaska Native/Native Hawaiian/ Pacific Islander: 8 (4.67%)     - Other: 8 (4.47%)   - Ethnicity     - Latina: 26 (14.53%)   - Year in school     - Freshman: 43 (24.39%)     - Sophomore: 41 (23.16%)     - Junior: 37 (20.90%)     - Senior: 44 (24.86%)   - Graduate students 12 (6.78%)   - Currently live:     - On-campus: 60 (35.50)     - Off-campus: 109 (64.50%) - Alcohol and drug use: 1.82 (SD: 1.46)   - Frequency of alcohol use in last 30 days: 1.69 (1.42)   - Frequency of getting drunk in last 6 months: 2.60 (1.36)   - Frequency of binge drinking in last 2 weeks: 2.02 (1.18)   - Any marijuana use in last 30 days: 84 (47.46%)   - Any drug use excluding marijuana in last 30 days: 37 (21.51%)   - Intimate partner violence - Total Composite Abuse Score (CAS) score: 28.62 (17.14) - Traumatic Brain Injury (TBI) related IPV: 49 (27.68%) - Reproductive Coercion (RC): 34 (18.99%)   - - Digital Abuse: 3.25 (2.14)     - Depression: 31.41 (19.57)     - Risk for suicide: 72 (41.4%) | - Alcohol and drug use (by frequency) - Safety behavior (by percentage of app features tried) - IPV by the following: - Composite Abuse Scale (CAS) score - Digital abuse (by 7 dichotomous questions) - Traumatic brain injury (4 question items) - Reproductive coercion (by 2 dichotomous question items) - Depression (by Center for Epidemiologic Studies Depression Scale Revised (CESD-R)) - Risk for suicide (by CESD Scale) - Decisional conflict and decision preparedness (by modified Decisional Conflict Scale (DCS) - Relationship between Safety behavior and IPV reduction (by mixed-effects regression analysis) |
| Debnam et al., 2021 [6] | - To investigate adolescents’ views on feasibility of the app for teen dating violence (TDV) intervention - To gather participants’ opinions on app’s assistance in dating violence | *Study type*   - Single-arm - Qualitative   *Period*   - Study: first 2 weeks of February, 2019 - App use: 60 – 75 minutes | All of the following:   - Enrolled in community college in northeastern state in USA - 14 -18 years of age - Agreed to participate in audio recorded focus group - Completed the online campus climate survey (at least half of the items on each scale) | Recruitment and orientation   - Adolescents recruited through local community-serving agencies in a small mid-Atlantic city - Research team members and local community members described the project to participants - Consent form provided to interested individuals, for parents or legal guardians to review and sign - App was introduced and used during focus group discussions   Data collection  Socio-demographic survey   - Topics covered: socio-demographic information - Analysis: descriptive     Focus groups   - 6 focus groups with approximately 4 participants (range: 2-8) per group - 60 – 75 minutes - Topics covered: - Language, format, and platform - Warning signs of dating abuse - Benefits and risks of using app to help a friend - Analysis: thematic   Interview   - A single-individual interview was conducted due to lack of participants - Topics covered: users’ perception and opinion on teen dating violence (TDV) and TDV preventing app - Analysis: thematic | - N = 23   Demographics   - Gender (N):   - Female: 15 (65.2%)   - Gender fluid: 4 (17.4%)   - Male: 4 (17.4%)      - Sexual orientation (N): - Heterosexual: 13 (56.5%) - Gay or lesbian: 3 (13.0%) - Bisexual: 5 (21.7%) - Other: 2 (8.7%) - Mean age (years): 16.43; SD: 1.34; Range: 14-18 - Ethnicity:   - White: 61%   - Black/African American: 30%   - Mixed: 9% | - Perception and feedback on the app and its features |
| Alhusen et al., 2015 [7] | - To have the app reviewed by friends of dating violence survivors | *Study type*   - Single-arm - Qualitative   *Period*   - Study: NR - App use: 20-40 minutes | All of the following:   - English speaking college students - 18-24 years of age - Reported having a friend who had experienced DV while in college | Recruitment and orientation   - Participants were recruited from four research sites:   - Johns Hopkins University, Arizona   - State University   - University of Missouri-Columbia   - Oregon Health   - Science University - Notification via:   - Craigslist   - Facebook   - Student listservs   - Campus fliers   - Word-of-mouth - App was provided in a pre-loaded iPod at the beginning of the focus group/interview   Data collection  Focus groups   - 6 group sessions - 3-6 participants per group) - 60-90 minutes long semi-structure interview guide was used - Topics covered: - App usability - Understandability - Appropriateness - Analysis: thematic   Interviews   - Approximately 60 minutes - Semi-structured - Topics covered: - App usability - Understandability - Appropriateness - Analysis: thematic | - Focus groups: N = 31 - Interviews: N = 8   Demographics (N = 31)   - Gender: 80.6% Female - Mean age (years): 20.84 years; Range: 18-24 - Ethnicity:   - White: 51.6%   - African American: 25.8%   - Asian: 9.7%   - Multiracial: 6.4%   - Hispanic: 3.2%   - Other: 3.2% | - Users’ perception of the app (usability, understandability, and appropriateness) |
| Lindsay et al., 2013 [8] | - To gather feedback on the evaluation of the decision aid app prototype from college-going females who are dating violence survivors | *Study type*   - Single-arm - Qualitative   *Period*   - Study: NR - App use: 60 – 90 minutes | All of the following:   - Enrolled in college in one of the four research sites; colleges located in the following states: - Arizona - Maryland - Missouri - Oregon - Female - English speaking - 18-25 years - Experienced dating violence in college | Recruitment and orientation   - Participant were recruited via:   - Craigslist   - Facebook   - Student listservs   - Campus flyers   - Word-of-mouth - Participants were contacted after screening by research team and were orientated about the app, the nature of the study, and their role in the study - US $25 given to participants, along with a dating violence resource sheet   Data collection  Focus groups   - 10 group session (2-7 participants per group) - Approximately 90 minutes - Semi-structured - Topics covered: - App usefulness - Understandability - Appropriateness - Comprehensiveness - Analysis: descriptive   Interview (for those who did not want to join in the focus groups)   - Approximately 60 minutes - Semi-structured - Topics covered: - App usefulness - Understandability - Appropriateness - Comprehensiveness - Analysis: descriptive | - Focus groups: N = 34 - Interviews: N = 4   Demographics (N = 38)   - Mean Age (in years): 21.26 (SD = 1.86) - Race/Ethnicity:   - White: 52.6%   - Hispanic/Latina: 23.7%   - African American: 7.9%   - Multiracial: 13.2%   - Other: 2.6% - Level at College:   - Freshman: 5.2%   - Sophomores: 13.2%   - Juniors: 21.1%   - Seniors: 28.9%   - Graduate level: 10.1% - Proportion of participants currently dating or in a relationship: 47% Proportion of participants who reported being in a previous relationship with an abusive female partner: almost 16% | - Users’ perception of the app (usability, understandability, and appropriateness, comprehensiveness) |
| uSafeUS (USA) | | | | | | |
| Potter et al., 2022 [9] | **Phase I**   - To gain input from key stakeholders to develop the app prototype   **Phase II**   - To test the app prototype   **Phase III**   - To test the app on iOS platform and to gather participants feedback   **Phase IV**   - To gather opinions from campus administrators and community agencies | *Study type*   - Single-arm - Qualitative   *Period*   - Study: NR - App use*:* NR | - Associated with any of the 5 selected pilot colleges in the USA in one or more of the following ways: - Students attending 2^nd^ and 4^th^ year colleges - College faculty and staff - Community agencies | Recruitment and orientation   - College students, staff, and faculty were engaged from five pilot sites through campus visits and electronic flyers - Community agencies were engaged - App overview was sent to participating schools along with promotional items   Data collection  Focus groups   - Approximately 60 minutes   **Phase I**  *Students, campus administrators, crisis center advocates, law enforcers*   - 6 group sessions - Topics covered: initial ideas for prototype app development - Analysis: thematic   **Phase II**  *Students, campus administrators, crisis center advocates*   - 5 focus groups - Topics covered:   - Testing of the app prototype in android phones   - Insights about on-campus promotion strategies - Analysis: thematic   **Phase III**  *Students*   - 5 focus groups - Topics covered:   - Beta testing on iOS platform   - Participants phone usage   - Suggestions on general and preventive app features   - App promotion in college campus - Analysis: thematic   **Phase IV**  *campus administrators, crisis center advocates, law enforcers*   - One focus group - Topics covered: improvements to resource information and preventive features - Analysis: thematic   *Students*   - Qualitative survey - Topics covered: app promotion to students - Analysis: thematic | **Phase I**   - Students, campus administrators, crisis center advocates, law enforcers: N = 60   **Phase II**   - Students, campus administrators, crisis center advocates: N = 50   **Phase III**   - Students: N = 50   **Phase IV**  Qualitative survey   - Students: N = 487 - Campus administrators and community agencies (e.g., police officers, crisis center advocates): N = 14     Demographics   - NR | **Phase I**   - Knowledge on app’s existence - Deciding factors for app download - Stakeholders’ input in app prototype development (all by focus group discussions)   **Phase II:**   - Stakeholders’ perception on app prototype (by focus group discussions)   **Phase III:**   - Mobile app usage - Feedback on app features and suggestions on app promotion (both by focus group discussions)   **Phase IV:**   - Administrators’ and agency representatives’ feedback on app’s resource information and prevention features (by focus group discussions) - App promotion to students (by survey) |
| Potter et al., 2020 [10] | - To understand the reasons for downloading, users’ perception, and usage of the app | *Study type*   - Cross-sectional - Quantitative   *Period*   - Study*:* First 2 weeks of February, 2019 - App use: ≤ 16 months | - Enrolled in Community college in a northeastern state in USA   AND   - Completed the online campus climate survey (at least half of the items on each scale) | Recruitment and orientation   - Participants were recruited from seven community college from a northeastern state via e-mail invitation - Participants had opportunity to win one of 50 US $50 gift cards - Participants provided with a list of campus and local resources including contact information for local rape crisis centre - App introduced to students in October 2017 via:   - Emails   - Flyers   - Social media   - Push notifications   - Visual display   - Promotional tabling event   - Classroom setting   - Informational meeting with campus representative   Data collection  Survey   - Approximately 25 minutes to complete - Topics covered: - Demography   Questionnaire   - Reasons for downloading app - Reasons for not downloading app - Confidence in helping a friend - Analysis: descriptive and inferential analysis (chi-square test) - Perception of safety | *College students*   - N = 1499   Demographics   - Female: 74.2% - Heard about the app: 20% - Downloaded the app: 24.7% of 20% - Age: NR | - uSafeUS app measure (by survey questions (check-marked)): - Reasons for downloading - Reasons for not downloading - Confidence in helping a friend - Recommending the app - Perception of safety (by 5-point Likert-type survey questions) |
| Bullying/High School Violence Prevention | | | | | | |
| +FORT (Stronger than Bullying) (Canada) | | | | | | |
| Ouellet-Morin et al., 2018 [11] | **Pilot study**   - To gain preliminary insights on the perceived usefulness, attractiveness, and acceptability of the app by youth, parents, and educators - To identify weaknesses of app to inform further development   **Empirical study 1**   - To gain victims’ perspectives on how the app may help them   **Empirical study 2**   - To examine the potential usefulness of the app in reducing bullying victimization over time   To determine if the observed findings are reflected in the data directly recorded by the app over the test period | *Study type*   - Single arm - Quasi-experimental (empirical study 2) - Mixed methods (empirical study 2)   *Period*   - Study: NR - App use: - Pilot study: 4 weeks - Empirical studies: 4 – 6 weeks - 17 participants allowed to use app for over 4 weeks due to logistical reasons | **Pilot study**   - Residing in Montréal, Canada   AND   - Youths 12 – 16 years of age   **Empirical study 1**  All of the following:   - Residing in Montréal, Canada - Youths 12 – 16 years of age - Victims of bullying (moderate-to-high level)   **Empirical study 2**  All of the following:   - Students from selected high schools in Québec, Canada - 12 – 16 years of age - All at the same grade level | **Pilot study**  *Youth, parents and educators*  Recruitment and orientation   - Participants invited to use app for 4 weeks   Data collection  Focus groups   - Post intervention - Two sessions: one for youth and one for parents and educators - Topics covered: - Perception of app - Most helpful features - Features needing improvement - Analysis: thematic   **Empirical study 1**  *Victimized youth*  Recruitment and orientation   - Youth recruited via online advertisements and presentations in two youth centers in Montréal, Canada - Participants included based on their score on the Multidimensional Peer-Victimization Scale (MPVS, 16 items) - Participants provided with an iPhone, where necessary - Oral parental consent given over phone prior to interview   Data collection  Interviews   - Approximately 60 min - Semi-structured - Topics covered: - Users’ experience and perception of app - Experiences of bullying prior to using app - Utility of app - Analysis: thematic   Questionnaire:   - Pre-post - Topic covered: bullying experiences - Analysis: inferential (ANOVA)   **Empirical study 2**  *Victimized youth*  Recruitment and orientation   - Participants recruited from one public and one private high school in Québec, Canada - Intervention group received app for 4 – 6 weeks - Control group did not receive the app   Data collection Questionnaires   - Pre-post - Topics covered: bullying experiences - Analysis: Inferential (ANOVA and generalized mixed model) | - N = 235 - Youth: N = 230 - Parents and educators: N = 5   **Pilot study**   - Youth: N = 8 - Parents and educators: N = 5   **Empirical study 1**   - Youth: N = 12   **Empirical study 2**   - Students (total): N = 210 - App users: N = 40 - Victims of moderate-to-high levels of bulling: N = 23 - Occasional victims (recruited due to low number of other victims recruited): N = 5 - Non-victims: N = 12 - Control: N = 170   Demographics  **Pilot study**   - Gender: NR - Age: NR   **Empirical study 1**   - Gender (N): - Female: 9 (75%) - Age: 12 – 16 years   **Empirical study 2**   - Gender (N): - App users - Female: 17 (42.5%) - Age: 12 – 16 years - Control - Gender: NR   Age: 12 – 16 years | **Pilot study**  *Youth, parents and educators*   - Perceived usefulness   **Empirical study 1**  *Youth*   - Perceived usefulness - Bullying victimization (by MPVS questionnaire)   **Empirical study 2**  *Youth*   - Bullying victimization (by MPVS questionnaire) - Agreement between questionnaire and app-recorded data (by questionnaire and app-recorded data) |
| uSafeHS (USA) | | | | | | |
| Potter et al., 2022 [12] | **Phase I** (wireframe development)   - To gather input to develop a prototype   **Phase II** (prototype development)   - To test the prototype and gather feedback   **Phase III** (pilot)   - To gather feedback on the final version and collect suggestion to promote user engagement | *Study type*   - Single-arm - Qualitative   *Period*  Study:   - Phase I: September 2019 - April 2020 (focus group) + 6 weeks for analysis - Phase II: May - August 2020 + 8 weeks for analysis - Phase III: October -December 2020 (8 weeks) - App use (Phase III): October-December, 2020 (8 weeks) | - Associated with any of the 7 selected public and private schools in New England in one or more of the following ways: - Students - School administrators and staff - Parents and guardians | Recruitment and orientation   - Project team worked with school administrators to recruit students - School administrators and teachers provided study details to participants in-person and via email - Students, school administrators, staff, parents and guardians for focus group and survey data were engaged from 13 high schools and pilot data were collected from seven high schools (both public and private) in New England   Data collection  **Phase I**  Focus groups:   - 40 - 60 minutes - 10 group sessions with students, 10 sessions with high school administrators and staff, 6 sessions with parents - Topics covered:   *Students*   - - Students’ concerns   - Supporting tools   - Suggestions for resources to promote healthy relationships and student safety   *School administrators, staff and parents*   - - Prevention needs within school community   - Tools and resources needed to support students - Analysis: thematic   Online Survey  *Students*   - Topics covered: - Phone brand - Time spent on phone - Preferred methods of communication - Mode of help-seeking in difficult situations - Current smartphone usage - Influential factors for downloading apps - Analysis: descriptive   **Phase II**  *SAB and FSAB*  Focus groups   - Biweekly from May- August 2020 - Topics covered: - Prototype app development - Feedback on app features - Feedback on administrator dashboard and strategies of app dissemination in school - Analysis: thematic   **Phase III**  *Students*  Aggregated data from administration dashboard   - Topics covered: app usage - Analysis: No statistical analysis done   Focus groups   - Biweekly group sessions conducted from May – August 2020 - Topics covered: - Suggestions to improve existing app features - Strategies to promote user engagement - Analysis: thematic   Online Survey  *Students*   - Topics covered: specific features of app - Analysis: descriptive | **Phase I**  Focus groups   - Students: N = 199 - High school administrators and staff: N = 68 - Parents: N = 26   Survey   - Students: N = 199   **Phase II**  Focus groups   - Student advisory board (SAB): N = 20 - Faculty and staff advisory board (FSAB): N = 9   **Phase III (8 week)**  *Students*   - App use: N = NR   (Usage data obtained from app’s  administrative dashboard)   - Focus groups: N = 14 - Online survey: N = 30   Demographics  NR | **Phase I**   - Technology use - Participants’ perspective on sexual violence preventive app and protective resources   **Phase II**   - Feedback on features from school community   **Phase III**   - App usage (by aggregated data from administration dashboard) - Users’ feedback on app features |
| Self-harm/ Suicide Prevention | | | | | | |
| BlueIce (UK) | | | | | | |
| - Grist et al., 2018 [13] - Stallard et al., 2018 [14] | - To assess the acceptability, use, and safety of BlueIce [13] - To assess the effects of BlueIce on self-harm and psychological functioning in young people [14] | *Study type*   - Single-arm - Mixed methods [13, 14]   *Period*   - Study: May – November 2016 (recruitment) [13, 14] - App use: 12 weeks - Familiarization: 2 weeks - After familiarization: 10 weeks [13, 14] | All of the following:   - Attending CAMHS (Child and Adolescent Mental Health Services) - Young people - 12 – 17 years of age - Currently or previously self-harmed [13, 14] | Recruitment and orientation   - Participants identified by their CAMHS clinician - Initial meeting at baseline, followed by a 2-week familiarization period and subsequent post-familiarization meeting (prior to an additional 10 weeks of app use) - Written consent obtained from participants aged 16 and older - Written parent consent and child assent obtained for participants under 16 years of age - BlueIce provided as an adjunct to face-to-face meeting s with CAMHS clinician [13, 14]   Data collection   - Pre-post - Baseline - Post-familiarization: 2-week - Post-use: 12-week [13, 14]   Interviews   - About 40 min - Semi-structured - Topics covered: participant’s perspectives on safety, acceptability, and usability of app - Analysis: thematic [13, 14]   Standardized questionnaires   - Topics covered:   - Depression   - Anxiety   - Behavior [13, 14] - Analysis: descriptive and inferential statistics [13, 14] | Semi-structured interviews  *Children and adolescents who self-harm (app users)*   - Baseline: N = 44 [13, 14] - Post-familiarization: N = 40 (90%) - Used app: 92% - Did not use app: 8% [13, 14] - Post-use (follow-up): N = 33 (82%) [13, 14]   Standardized questionnaires   - Baseline: - App users: N = 30 – 32 - Parents (Revised Child Anxiety and Depression Score (RCADS) and Strengths and Difficulties Questionnaire (SDQ): N = 18 [13, 14] - Post-use: - App users: N = 30 – 32 - Parents (RCADS and SDQ): N = 10 – 13 [13, 14]   Demographics (N = 44 app users)   - Female: 90% [13, 14] - Mean age (SD): 15.98 years (1.37) [13, 14]   Psychological characteristics:   - Self-harmed at least once in 4 weeks prior to starting trial: 30 (68%) [13, 14] [13, 14] - Probable depression (based on MFQ score): 42 (95%) [13, 14] - Screening positive for at least 1 anxiety disorder (based on RCADS): 37 (84%) [13, 14] - Probable emotional disorder (based on SDQ score): 37 (84%) [13, 14] - Rated by parent as having depression (based on RCADS score): 16/17 (94%) - Rated by parent as having significant emotional problems (based on SDQ score): 16 / 18 (88%) [13, 14] | - Acceptability (by interview and 10-point Likert scale) [13, 14] - Usability ( 10-point Likert scale) [13, 14] - Safety (5-point Likert scale) [13, 14] - Depression (by MFQ) [13, 14] - Anxiety (by RCADS) [13, 14] - Behavior (by SDQ) [13, 14] - Self-harm (by clinical records and self-reporting) [13, 14] |
| *iBobbly (Australia)* | | | | | | |
| Tighe et al., 2020 [15] | - To determine the pilot usage and acceptability of the iBobbly suicide prevention app | *Study type*   - RCT - Mixed methods   *Period*   - Study: 2015 - App use: 6 weeks | All of the following:   - Aboriginal and Torres Strait Islander - Residing close to Broome, Western Australia - 8 – 35 years of age (18 – 25 initially targeted) - Previous participation in iBobbly app - Had moderate scores for depression or psychological distress - With or without current suicidal ideations | Recruitment and orientation   - Participants were a subset of a larger cohort study [16]   Data collection  Survey (General internet use)   - Focused on general internet use - Topics covered: - Frequency and duration of general internet use - Types of devices used online - Types of online activities - Analysis: descriptive statistics   Interviews   - Semi-structured - Topics covered: experience of using iBobbly app - Analysis: descriptive and inductive thematic analysis   Usage data   - Pre-post - Impact of usage time on suicidal ideation, depression, and psychological distress - Analysis: descriptive and inferential statistics (regression analysis) | *Young Aboriginal and Torres Strait Islander with depression or psychological distress*  Interviews (iBobbly app)   - N = 13   Usage data (iBobbly app)   - N = 40   Demographics (N=13)  Survey and interviews   - Female: 10 (77%) - Mean age (years): 24.15; range: 19 – 29; SD: 4.7 - Employment: - Employed: 7 (54%) - Student: 6 (46%) | - General internet use (The Measure of Technology Use survey) - App usage (by automatic downloading from mobile devices) - Time spent logged onto the app - Time spent on each self-assessment (3 in total) - Time spent on each content module (3 in total) - Number of times emergency help button pressed - Users’ perception - Suicidal ideation (by Depressive Symptom Inventory – Suicidality Subscale (DSI-SS) - Depression (by Patient Health Questionnaire 9 (PHQ-9) - Psychological distress (by Kessler Psychological Distress   Scale (K10) |
| Tighe et al., 2017 [16] | - To assess the impact of iBobbly app on suicidal ideation, depression, psychological distress and impulsivity in Aboriginal and Torres Strait Islander youth in remote Australia - To pilot the implementation of an RCT in Aboriginal and Torres Strait Islander communities with a focus on suicide prevention | *Study type*   - RCT - Quantitative   *Period*   - Study: September 2013 – March 2015 - App use: 6 weeks | - Aboriginal and Torres Strait Islander Australians - 18 – 35 years of age - A score of > 10 on PHQ-9 or a score of ≥ 25 on K10 scale - Had suicidal thoughts in last 2 weeks   Exclusion   - Individuals who experience schizophrenia, psychosis, or active intent to suicide | Recruitment and orientation   - Recruitment facilitated by collaboration with Men’s Outreach Service in Broome, Western Australia, which manage an Aboriginal and Torres Strait Islander youth suicide prevention project in partnership with Aboriginal and Torres Strait Islander community volunteers - Facebook page, posters and flyers in medical centers, information sessions with health professionals - Research promotion by Aboriginal and Torres Strait Islander staff - 15-minute training session, include setting password for secure access - Android tablet with the pre-installed app provided to participants   Data collection   - Two-arm randomized controlled trial - One intervention group (6 weeks) - One 6-week waitlist control group (participants received the app for the 6 weeks following the wait period) - Pre-post - Baseline - 3 weeks - 9 weeks - Waitlist group was assessed at 9-week and 12-week timepoints - Analysis: Inferential (mixed-model repeated measures PFAS) | *Young, Aboriginal and Torres Strait Islander with suicidal ideations*   - N = 61 - Intervention: N = 31 - Waitlist: N = 30 - Usage data: N = 40 - No data provided: N = 21   Demographics (N = 61)   - Intervention - Female: 20 (65%) - Mean age (SD): 27.48 (9.54) - Aboriginal or Torres Strait Islander origin - Aboriginal: 30 (97%) - Torres Strait Islander: 1 (3%) - Both Aboriginal and Torres Strait Islander: 0 - Neither aboriginal or Torres Strait Islander: 0 - Employment status - Employed full-time: 10 (32%) - Employed part-time: 5 (16%) - Unemployed/ looking for work: 11 (35%) - Not in the labor force: 5 (16%) - Main activity (if not in the labor force))   - - - Home duties/ caring for children: 12 (39%) - Schooling level completed - Some secondary: 5 (16%) - Four years of secondary: 17 (55%) - Six years of secondary: 9 (29%) - Tertiary schooling: - None: 25 (81%) - Control - Female: 19 (63%) - Mean age (SD): 24.97 (6.28) - Aboriginal or Torres Strait Islander origin - Aboriginal: 25 (83%) - Torres Strait Islander: 0 - Both Aboriginal and Torres Strait Islander: 1 (3%) - Neither aboriginal or Torres Strait Islander: 4 (13%) - Employment status - Employed full-time: 9 (30%) - Employed part-time: 4 (13%) - Unemployed/ looking for work: 12 (40%) - Not in the labor force: 3 (10%) - Main activity (if not in the labor force)   -Home duties/caring for children: 9 (30%)   - Schooling level completed - Some secondary: 0 - Four years of secondary: 11 (37%) - Six years of secondary: 19 (63%) - Tertiary schooling: - None: 23 (77%) - Referrers - Aboriginal and Torres Strait Islander health professionals: 75% - Mainstream mental health services: 25% | - App usage (by automatic downloading from mobile devices) - Suicidal ideation (by DSI-SS - Depression (by PHQ-9) - Psychological distress (by K10) - Impulsivity (by Barratt Impulsivity Scale (BIS-11)) |

**References**

1. Sheoran B, Silva CL, Lykens JE, Gamedze L, Williams S, Ford JV, et al. YTH StreetConnect: Development and Usability of a Mobile App for Homeless and Unstably Housed Youth. JMIR mHealth and uHealth. 2016;4(3):e82. [doi: [10.2196/mhealth.5168](https://doi.org/10.2196/mhealth.5168)]

2. Blayney JA, Jenzer T, Read JP, Livingston JA, Testa M. Enlisting friends to reduce sexual victimization risk: There's an app for that… but nobody uses it. J Am Coll Health. 2018;66(8):767-73. [doi: [10.1080/07448481.2018.1446439](https://doi.org/10.1080/07448481.2018.1446439)]

3. Navarro-Pérez JJ, Oliver A, Carbonell Á, Schneider BH. Effectiveness of a mobile app intervention to prevent dating violence in residential child care. Psychosoc Interv. 2020;29(2):59-66. [doi: [10.5093/pi2020a3](https://doi.org/10.5093/pi2020a3)]

4. Navarro-Pérez J-J, Carbonell Á, Oliver A. The effectiveness of a psycho-educational app to reduce sexist attitudes in adolescents. Rev Psicodidáct (English ed.). 2019;24(1):9-16. [doi: [10.1016/j.psicod.2018.07.002](https://doi.org/10.1016/j.psicod.2018.07.002)]

5. Glass N, Clough A, Messing J, Bloom T, Brown M, Eden K, et al. Longitudinal Impact of the myPlan App on Health and Safety Among College Women Experiencing Partner Violence. J Interpers Violence. 2022;37(13-14). [doi: [10.1177/0886260521991880](https://doi.org/10.1177/0886260521991880)]

6. Debnam K, Kumodzi T. Adolescent Perceptions of an Interactive Mobile Application to Respond to Teen Dating Violence. J Interpers Violence. 2021;36(13-14). [doi: [10.1177/0886260518821455](https://doi.org/10.1177/0886260518821455)]

7. Alhusen J, Bloom T, Clough A, Glass N. Development of the MyPlan Safety Decision App with Friends of College Women in Abusive Dating Relationships. J Technol Hum Serv. 2015;33(3):263-82. [doi: [10.1080/15228835.2015.1037414](https://doi.org/10.1080/15228835.2015.1037414)]

8. Lindsay M, Messing JT, Thaller J, Baldwin A, Clough A, Bloom T, et al. Survivor Feedback on a Safety Decision Aid Smartphone Application for College-Age Women in Abusive Relationships. J Technol Hum Serv. 2013;31(4):368-88. [doi: [10.1080/15228835.2013.861784](https://doi.org/10.1080/15228835.2013.861784)]

9. Potter SJ, Moschella EA, Demers JM, Lynch M. Using Mobile Technology to Enhance College Sexual Violence Response, Prevention, and Risk Reduction Efforts. J Technol Hum Serv. 2022;40(1):25-46. [doi: [10.1080/15228835.2021.1929665](https://doi.org/10.1080/15228835.2021.1929665)]

10. Potter SJ, Moschella EA, Smith D, Draper N. Exploring the Usage of a Violence Prevention and Response App Among Community College Students. Health Educ Behav. 2020;47(1_suppl):44S-53S. [doi: [10.1177/1090198120910995](https://doi.org/10.1177/1090198120910995)]

11. Ouellet-Morin I, Robitaille M-P. Stronger than Bullying, a mobile application for victims of bullying: Development and initial steps toward validation. In: Campbell M, Bauman S, editors. Reducing Cyberbullying in Schools. Academic Press; 2018. p. 159-74.

12. Potter SJ, Moschella-Smith EA, Lynch M. Building a high school violence prevention app to educate and protect students. J Res Technol Educ. 2022:1-19. [doi: [10.1080/15391523.2022.2110336](https://doi.org/10.1080/15391523.2022.2110336)]

13. Grist R, Porter J, Stallard P. Acceptability, Use, and Safety of a Mobile Phone App (BlueIce) for Young People Who Self-Harm: Qualitative Study of Service Users’ Experience. JMIR Mental Health. 2018;5(1):e16. [doi: [10.2196/mental.8779](https://doi.org/10.2196/mental.8779)]

14. Stallard P, Porter J, Grist R. A Smartphone App (BlueIce) for Young People Who Self-Harm: Open Phase 1 Pre-Post Trial. JMIR mHealth and uHealth. 2018;6(1):e32. [doi: [10.2196/mhealth.8917](https://doi.org/10.2196/mhealth.8917)]

15. Tighe J, Shand F, Mckay K, Mcalister T-J, Mackinnon A, Christensen H. Usage and Acceptability of the iBobbly App: Pilot Trial for Suicide Prevention in Aboriginal and Torres Strait Islander Youth. JMIR Mental Health. 2020;7(12):e14296. [doi: [10.2196/14296](https://doi.org/10.2196/14296)]

16. Tighe J, Shand F, Ridani R, Mackinnon A, De La Mata N, Christensen H. Ibobbly mobile health intervention for suicide prevention in Australian Indigenous youth: a pilot randomised controlled trial. BMJ Open. 2017;7(1):e013518. [doi: [10.1136/bmjopen-2016-013518](https://doi.org/10.1136/bmjopen-2016-013518)]
