# Supplementary material for: Mobile Apps for the Personal Safety of At-Risk Children and Youth: Scoping Review
Source: JMIR Mhealth Uhealth. 2024 Nov 5;12:e58127. doi: 10.2196/58127 (PMC11576608; doi:10.2196/58127)
Supplement: Multimedia Appendix 5 [file mhealth_v12i1e58127_app5.docx]

## Multimedia Appendix 5. Findings of studies evaluating personal safety apps for youth

| First author, year | Aim | Findings | Facilitators, barriers, and limitations | Conclusions and Recommendations for app development |
| --- | --- | --- | --- | --- |
| Homeless Youth Support | | | | |
| YTH StreetConnect + YTH StreetConnect Pro (USA) | | | | |
| - Sheoran et al., 2016 [1] | - To conduct usability and feasibility testing of the app among homeless and unstably housed (H/UH) youth and local technical experts who serve H/UH youth | - Users’ experiences   *Youth*   - App was well received (“Intuitive, fun and easy to use”) - Map feature considered to be essential - App thought of as a blend of social media and app function (e.g. Google and Yelp)   *Service providers*   - App perceived to be “nice, clean and easy to read”   *Youth and service providers*   - App perceived as accessible and appropriate for locating services - App provided confidential access to resources - App acted as a supplementary to in-person interaction between providers and youth - Feasibility   *Youth*   - High likelihood of repeated use of the app   *Service providers*   - Useful; “statistics and resource information were most useful” - Implemented changes to app - Resource information and map on one screen - Peer rating system - Service icons - Online forum for youth to share experience | *Facilitators to app use*   - Easy to use - Visual enhancement   *Barriers to app use*   - NR   *App limitations*   - Limited resource features | *Conclusions*   - Bases on users’ perceptions, app is a useful tool for streamlining all services for H/UH youth and their service providers   *Recommendations*  *Youth*   - “Home” button on all screens - Feature that shows number of available beds at local shelters   *Study Limitations*   - Small sample size - Period of app engagement not specified - No information on size and length of focus groups - No validated outcome measures used |
| Dating/Sexual violence prevention | | | | |
| Circle of 6 (USA) | | | | |
| - Blayney et al., 2018 [2] | - To assess the feasibility and acceptability of Co6 among college women who drink alcohol - To assess how the app is perceived as a sexual violence risk reduction tool among young women | - Helping others:   - Protective behavior:     - Nearly three times more protective behavior observed towards friends compared to strangers - Feasibility and acceptability:   - Friends & prevention:     - Proportion who talked with friends about the app: 70%     - Users’ average number of friends:       - Spoken to about the app: 3.86 (SD: 1.12); range: 2–5       - Added in the app: 4.79 (SD: 1.34); range: 2–6   - Number of users that informed their friends about adding them to app circle: 35 (80%)   - App use and risk context:     - Average app use: once over 2-month period     - Low engagement with app, considering high frequency of drinking     - Users’ average number of drinking occasions over 2 months: 15.16 (SD: 6.92); range: 2–40     - Proportion who used the app at least once in a drinking context: 75% (N = 33)     - Average frequency of app use in drinking context: 1.09 (SD: 1.34); range: 0-5     - Most common time for app use: 9 pm - 3 am - App’s subjective value by users (M): - App’s utility score: M = 5.20 (SD: 2.60); range: 1–10 (higher score reflects higher subjective value) - Feeling of safety from sexual violence while using the app: M = 5.26 (SD: 2.85); range: 1–10 (higher score reflects higher degree of safety perception) - Number of participants that remained neutral on app referral: 5.64 (SD: 3.19); range: 1-10 (higher score reflects higher referral intension) - Impression of the app:   - Positive themes:     - Proportion who felt “easy connection with friends”: 48%     - Proportion who liked the app features, especially “Location” feature: 36%     - Proportion who thought the app was “good in theory”: 27%   - Negative themes:     - Proportion who perceived the app as unnecessary: 55%     - Proportion who felt uncomfortable with group messaging: 32%     - Proportion who felt the app had limited context for use: 23% - Involvement in risk context:   - Sexual victimization:     - Proportion who reported sexual victimization at follow-up: 23% (N = 10)     - Level of violence:       - Unwanted sexual contact: 5 (50%)       - Attempted rape: 2 (20%)       - Completed rape: 3 (30%)     - Relation of perpetrators to users (N = 9):       - Strangers: 3 (33%)       - Friends/Acquaintances: 4 (44%)       - Boyfriends: 2 (22%)     - Place of occurrence (N = 9):       - Bars: 4 (44%)       - Parties/social gathering: 4 (44)       - Date: 1 (11)%     - Average number of alcoholic drinks consumed prior to assault: 5.78 (SD: 3.87); range: 1-12     - Average feeling of intoxication on a scale of 1-10: 4.33 (SD: 1.00); range: 2-5     - Average number of alcoholic drinks consumed by perpetrator (by participants’ estimation): 5.56 (SD: 3.17); range: 0-10     - Average feeling of intoxication of perpetrators on a scale of 1-10: 4.44 (SD: 1.74); range: 1-7 - App use was low due to perceived redundancy with existing smart phone features and college women’s discomfort with group messaging | *Facilitators to app use*   - App leverages personal relationships in risk reduction - Theoretical and practical appeal of app - Easy mode of connectivity - Appealing app features   *Barriers*   - Heavy alcohol consumption prevented users from using the app - Perception of app as an “emergency only” option   *App limitations*   - App perceived as redundant, considering other existing smartphone features - The fast pace of technological innovations may result in app upgrades quickly becoming outdated, reducing the likelihood of app use | *Conclusions*   - Bases on users’ perceptions, app is not to be useful for the practical needs of college women   *Recommendations*   - NR   *Study Limitation*   - Small sample size - Retrospective reporting susceptible to memory bias - Short follow-up period |
| Liad@s (Spain) | | | | |
| - Navarro-Pérez et al., 2020 [3] | - To determine the effectiveness of the Liad@s app in addressing three variables associated with dating violence in adolescents: sexism, romantic love myths, and ambivalence and prejudice towards men - To determine the acceptability of the app | - Sexism: - Statistically significant reduction in hostile sexism and benevolent sexism post intervention relative to pre intervention (P=.009 and P<.001, respectively) - Greater reduction in hostile sexism and benevolent sexism in intervention group compared to control group - Ambivalence towards men: - Statistically significant reduction in ambivalence towards men post intervention relative to pre intervention (P=.02) - Distortions about romantic love (love myths): - Statistically significant reduction in distortions of romantic love post intervention relative to pre intervention (P<.001) - Statistically significant difference in distortions of romantic love in intervention group compared to control group (P<0.001); (no change in control group) - No statistically significant gender differences in sexism or myths about romantic love - Statistically significant decrease in hostile sexism as age increases (P=.04) - Marginally significant decrease in paternal resentment with age (P=.06) - Acceptability and users’ experiences: - Proportion of adolescents who felt that the app showed them other points of view: 68.6% - Proportion of adolescents who felt that the app helped them to gain greater maturity in the area and made them try to improve: 22.9% - Proportion of adolescents who felt that the app did not have any impact on the way they related to other people: 8.6% | *Facilitators*   - NR   *Barriers to app use*   - NR   *App limitations*   - NR | *Conclusions*   - App was effective in reducing sexism in adolescents and in promoting healthy romantic relationships   *Recommendations*   - NR   *Limitations*   - Possible gender bias in using the Ambivalent Sexism Inventory, as focused on women only, but was used by both males and females - Normative and operational differences among the centers resulted in differences in the use of mobile phones |
| - Navarro-Pérez et al., 2019 [4] | - To evaluate the effectiveness of Liad@s app in reducing sexist beliefs and attitudes in adolescents | - Sexism - Statistically significant reduction in hostile sexism and benevolent sexism post intervention relative to pre intervention (P<.001 in both cases) - No change observed in the control group | *Facilitators*   - NR   *Barriers to app use*   - NR   *App limitations*   - NR | *Conclusions*   - App was effective in reducing sexism in adolescents   *Recommendations*   - NR   *Limitations*   - Participating schools needed to have information and communications technology - Self-reporting of outcomes prone to social desirability bias - Short follow-up period |
| myPlan (USA) | | | | |
| - Glass et al., 2022 [5] | - To compare difference in change over time in health and safety outcomes among female college students who used myPlan app compared to those who used usual web based safety planning resources | - Alcohol and drug use - Statistically significant pre-post reduction in frequency of getting drunk; no statistically significant difference between intervention and control groups - Non-statistically significant pre-post reduction in frequency of alcohol use, getting drunk, binge drinking, and non-marijuana drug use in both the intervention and control groups - Non-statistically significant pre-post increase in marijuana use in both groups - Safety behavior:   - Safety behavior improved over time, although improvement did not differ between groups - Intimate partner violence (IPV):   - Statistically significant decrease over time in both groups in Composite Abuse Scale score ( P<.001) traumatic brain injury score (P<.001), digital abuse score ( P<.001) and reproductive coercion (RC) score (P=.010)   - Statistically significantly greater reduction in RC in intervention group compared to control group (P=.019)   - Statistically significant improvement in depression over time for both groups (P<.001)   - Observed change over time did not differ between groups (P=.65) - Risk for suicide:   - Statistically significant reduction in suicide risk among intervention group over 12 months (P=.048) - Decisional conflict and decision preparedness:   - Immediate improvements in all subscales in both groups; greater change in total Decisional Conflict Scale score in intervention group than control group   - Statistically significantly more positive response on “helped you think about which risks and benefits of each safety option are most important to you” in intervention group compared to control group (P=.038)   - Statistically significantly greater number of positive responses on “prepared to make better safety decisions” by intervention group compared to control group at 12-months (P=.02) - Relationship between safety behaviors and IPV reduction   - Statistically significant association between number of safety behaviors tried and IPV reduction (P<.001) in intervention group only   - Statistically significant association between the increase in safety behaviors tried and the reduction in IPV from baseline to 12-months for the intervention group (P=.039)   - Similar number of safety behaviors tried in both groups (7.78 vs 7.56; P=.685)   - Similar proportion of safety behaviors found to be helpful in both groups (70.50 vs 68.77; P=.58) | *Facilitators to app use:*   - NR   *Barriers to app use*   - NR   *App limitations*   - NR | *Conclusions*   - Findings did not support the effectiveness of myPlan over usual safety planning on all outcomes - Study supports usefulness of technology based safety planning for young women who recently experienced IPV   *Recommendations*   - NR   *Study limitations:*   - Limited generalizability - Baseline survey questions may have acted as intervention, therefore, chances of bias - Absence of true control group (due to ethical reasons) |
| - Debnam et al., 2021 [6] | - To investigate adolescents’ views on feasibility of the app for teen dating violence (TDV) intervention - To gather feedback on how app may be improved to better assist adolescents who experience TDV | - Perception and feedback on the app and its features: - Careful and thoughtful inclusion of diverse adolescents was suggested - Thoughtful about males being the victims, as “women can be perpetrators too” - Intolerance of gender-conforming language in the app, which targeted females only - Criteria of the person/organization teens tend to go to for help: - Trustworthy and keeps confidentiality - Understanding - Implemented recommendations: - LGBTQ version developed and released based on feedback from participants | *Facilitators to app use*   - NR   *Barriers to app use*   - App targeted only females as victims of sexual violence   *App limitations*   - Lack of focus on safety dilemmas faced by youth such as: - Emotional or psychological abuse - Power imbalance induced by age difference | *Conclusions*   - Based on users’ perceptions, app could be modified to be more inclusive to users of diverse sexual orientations; LGBTQ version developed and released resulting from participants’ feedback   *Recommendations*   - Dating violence (DV) reporting options - Panic button option - Bystander-focused intervention for reducing DV   *Study limitations*   - Short study period (2 weeks) - No data was collected about participants’ exposure to TDV or violence in their home or community - Possible perception bias due to their own experiences with the TDV - Possible readability or comprehension challenges with app content |
| - Alhusen et al., 2015 [7] | - To have the app reviewed by friends of dating violence survivors | - Users’ perception: - Usefulness:   - App could be beneficial, effective, and resourceful for dealing with DV   - App features were informative, especially the “Myth” section   - App helped friends to understand abusive relationships better - Understandability:   - App was easy to understand navigate   - App helped participants to better understand the severity of violence, and to identify resource options and ways to intervene   - Positive feedback provided on graphical display and danger assessment tool - Appropriateness:   - App would be appropriate and informative in efforts to help friends   - Educational content helpful for victims’ friends who may not be ready to talk about sexual violence directly | *Facilitators to app use*   - The option to not discuss the sexual violence issue face to face (privacy and accessibility) - App removes potential for the individual to be judged - Incorporation of best IPV practices, evidence-based risk assessment, individualized feedback, and survivor-centered approaches   *Barriers to app use*   - NR   *App limitations*   - NR | *Conclusions*   - Based on users’ perceptions, app is a clear, helpful, and valuable tool to assist DV victims’ friends in helping them   *Recommendations*   - NR   *Study limitations*   - Limited information gathered on effective ways to disseminate app |
| - Lindsay et al., 2013 [8] | To gather feedback on the evaluation of the decision aid app prototype from college-going females who are dating violence survivors | - Users’ perception of the app: - Usefulness: - App found to be “useful, innovative and effective” in conveying information regarding dating violence and relationship safety - Perceived as being “a mirror” and “non judgmental” or a “non pushy” way to help self-assess someone’s relationship - Information on healthy relationship found useful - Mixed feedback on “My priorities” feature provided - Positive feedback on instant and color coded “My safety” option - “My Plan” option perceived as resourceful and helpful for personal safety, especially for those who do not know where to seek help - Understandability: - App perceived as “understandable and easy to navigate” - Contrasting views given on the volume of information on app   Appropriateness:   - Appropriate tool to assess dating relationship, especially for those who are not ready to address the crisis - Possibility that users who may not report true level of violence to the app may have a false sense of security - Accessing app preferred to going to others for help   Comprehensiveness:   - App viewed as “thorough”, “straightforward” and relevant - Implemented recommendations - Option to ideas on “My Plan” - Information on the roles of police - Additional information on emotional abuse | *Facilitators to app use*   - “Non judgmental and non pushy” way of helping participants   *Barriers to app use*   - Large volume of information in app was overwhelming to some - Women may not access the app safely without their stalker partner knowing   *App limitations*   - Lack of specialized resources for women at different stages of readiness in terms of their safety decision | *Conclusions*   - Based on users’ perceptions, app is a useful, understandable, appropriate, and comprehensive decision aid for college women in unsafe relationships   *Recommendations*   - Ambiguous name and branding - An easy “delete” option   *Study limitations*   - Study period and duration of app use not clearly stated - Group setting may have made disagreeing difficult for some participants |
| uSafeUS (USA) | | | | |
| - Potter et al., 2022 [9] | **Phase I**   - To gain input from key stakeholders to develop the app prototype   **Phase II**   - To test the app prototype   **Phase III**   - To test the app on iOS platform and to gather participants feedback   **Phase IV**   - To gather opinions from campus administrators and community agencies | **Phase I**   - Knowledge on app’s existence:   *Students*   - Learned about mobile apps through:   - Social and professional network   - Social media   - Advertising - Factors influencing app download in general:   - Cost of the app   - Storage space and data usage   - Ease of use (e.g., navigation)   - Restrictions and permissions requests (e.g., access to location, privacy settings)   - Reviews and ratings from fellow or previous users   - Informational content - Stakeholders’ input in app prototype development:   *Students, campus administrators, crisis center advocates, law enforcers*   - Proportion of focus group participants that agreed on the need of mobile apps to protect against sexual violence: 75% - Participants had positive perceptions of app’s sexual violence-related resources and supporting options - Factors that predominantly contributed to participants’ higher app-downloading:   - - Longer duration spent on campus or work     - Ease of navigation of the app - The majority of graduate students, (particularly older students) as well as commuter students, indicated that they did not perceive this app as being applicable or relevant to their needs - Implemented recommendations   - Option to use the app to text a friend during uncomfortable situations   - Option to quickly escape the app   **Phase II**   - Stakeholders’ perception on app prototype:   *Students, campus administrators, crisis center advocates*   - Implemented recommendations - “Expect Me” feature modified to enable GPS location sending to trusted contacts - Anonymous reporting option incorporated into the post-assault informational resources   **Phase III**   - Feedback on app features and suggestions on app promotion:   *Students*   - Used the Beta version of the app (refined following Phase II) on the iOS platform - Found the explanatory video for first time users cumbersome and suggested using dialogue boxes with brief instructions that would pop up - Participants suggested collaborating with first-year students to promote the app features, using promotional items/gifts to draw attention to the app   **Phase IV**  *Campus administrators, crisis center advocates, law*  *enforcers*   - Feedback on app’s resource information and prevention features - Agreed that resource information survivor-friendly and trauma-informed - Found app to be easy to access and necessary to help survivors after an assault - Suggested that links to external resource websites be directly accessible in app, rather than links that direct them to webpages in external apps such as Safari or Google Chrome   *Students*   - Perceived the app as “easy to access” and “necessary for survivors” in the aftermath of an sexual violence assault - Proportion of students (of N = 487) who believed on- and off-campus flyers would help with awareness: 51.5% - Proportion of students ( of N = 251) who thought the app should be discussed in class: 10% | *Facilitators to app use*   - Customizable app functions - Inclusion of educational and off-campus resources   *Barriers to app use*   - NR   *App limitations*   - NR | *Conclusions*   - Based on users’ perceptions, the app and its accompanying administrator dashboard provide “comprehensive prevention” (primary, secondary, and tertiary) against sexual violence for college students   *Recommendations*   - Including links directly within the app instead of being redirected to search engines to open external websites (suggested by Crisis center advocates)   *Study limitations*   - Possible researcher bias in data review and analysis, as two of the authors were creators of the app |
| - Potter et al., 2020 [10] | - To understand the reasons for downloading, users’ perception, and usage of the app | - uSafeUS app measure - Proportion who heard about the app: 20% (N = 288) - Proportion who downloaded the app among those who heard of it: 24.7% (N = 71), (app download range: 1.6% - 7.9%) - Strongly significant association between female gender and the number of individuals who downloaded the app (P<.001) - Significant association between downloading the app to help a friend and young age (P<.05) - Significant association between downloading the app for resource information feature and female gender (P<.05)      - Perception of safety - Significant association between perception of safety from sexual violence and app download (P<.05) - Significant association between receiving campus sexual violence information from college and downloading app download (P<.05) - Proportion who downloaded the app who felt confident that it would provide sufficient resources to help them support an assaulted friend: over 90% (N = 67) | *Facilitators to app use*   - Prevention and resource features - Young age and female gender   *Barriers to app use*   - Lack of information about the app   *App limitations*   - NR | *Conclusions*   - Based on users’ perceptions, app could act as a promising mechanism to deliver protection and response information to college-aged population about sexual violence   *Recommendations*   - NR   *Study limitations*   - Not clear if the students downloaded the app due to school’s promotion |
| Bullying/High School Violence prevention | | | | |
| +FORT: Stronger than Bullying (Canada) | | | | |
| - Ouellet-Morin et al., 2018 [11] | **Pilot study**   - To gain preliminary insights on the perceived usefulness, attractiveness, and acceptability of the app by youth, parents, and educators - To identify weaknesses of app to inform further development   **Empirical study 1**   - To gain victims’ perspectives on how the app may help them   **Empirical study 2**   - To examine the potential usefulness of the app in reducing bullying victimization over time   To determine if the observed findings are reflected in the data directly recorded by the app over the test period | **Pilot study**  *Youth, parents and educators*   - Perceived usefulness - Beneficial for victims - App would not jeopardize or conflict with existing services - Could learn from the app   **Empirical study 1**  *Youth*   - Perceived usefulness - Helped youth to have “more precise and realistic views” of their bullying experiences, including the setting, nature, and frequency - Helped youth reveal their experiences to someone they trusted - The two most helpful app functions (“Strategies” and “My Journal”) were associated with the following problem-solving techniques: - Viewing their bullying experiences more objectively - Adopting more effective strategies to respond to bullying problems - Proportion who initially refused to disclose their experiences: 100% - Proportion who wanted to express their experience after 4 weeks of using the app: 83% - Motivated youth to end bullying - Felt more empowered after using app - Bullying victimization - Statistically significant decrease in victimization (P=.001)     *Impact on personal safety*  **Empirical study 2**  *Students*   - Bullying victimization - Proportion that experienced cyberbullying at least once over the study period (35%) - Most of these individuals (86%) were cyberbullied only once - Statistically significant reduction (2-fold) in bullying victimization in victims (P<.001) and victims including occasional victims (P<.001) - The reduction in victimization was 16 times greater for victims compared to the control group - No statistically significant difference in bullying victimization observed for non-victims - Agreement between questionnaire and app-recorded data - Differences in the initial level of bullying victimization recorded by the app for victims compared to non-victims, indicating that the app can distinguish between victims and non-victims - Bullying levels decreased over time, particularly between weeks 4 and 5 | *Facilitators to app use*   - Confidentiality of app (e.g., password-protected) - Privacy of app, which facilitated disclosure of their experiences without feeling judged or pitied - Numerous and diverse strategies - Virtual map - Videos - Regular evaluation of bullying experiences - High level of accessibility of app and online resources   *Barriers to app use*   - NR   *App limitations*   - NR | *Conclusions*   - Based on users’ perceptions, app is a useful tool to youth who are victims of bullying, adults working with youth, and parents - App resulted in reductions in bullying victimization over time   *Recommendations*   - Enhanced visual presentation of graphs   *Study limitations*   - Small sample size - Short follow-up period - Possible social desirability bias - Possible recall error - No validated outcome measures used |
| USafeHS (USA) | | | | |
| - Potter et al., 2022 [12] | **Phase I:** Wireframe development   - To gather input to develop a prototype   **Phase II**: Prototype Development   - To test the prototype and gather feedback   **Phase III:**  Pilot   - To gather feedback on the final version and collect suggestion to promote user engagement | **Phase I**  *Students*   - Technology use - Proportion of iPhone users: 86.4% - Proportion who spent about 3 hours daily on phone: 75% - Proportion who used mobile phones for:   - Social media: 97%   - Communication via text messages: 96%   - Music: 94.9%   - Entertainment: 90.4%   - Games: 65.7% - Preferred communication mode:   - Texting: 97.5%   - Calling: 83.8%   - Online channels: 72.2%   - Video chat: 72.2% - Places/sources that students would seek help from when faced with a problem: - Friend: 86.9% - Parents or other family member: 77.4% - A professional: 27.1% - National resources: 8% - Perspective on sexual violence preventive app and protective resources:   *Students*:   - Showed interest in learning about relationship topics from reliable sources - Were interested in learning about red and green flags of relationships - Were interested about learning about emotional regulation, communication, and boundary setting - Expected “gamified”/interactive, reward based educational contents and practical features, online and school resources, and safe reporting features to be included in the app   *School administrators, staff and parents:*   - Felt a need for centrally accessible preventive educational resources, and safety tools for both students and the school community - Felt that the app should cover communication skills, boundary setting skills, skills related to helping a friend or self, and topics related to school violence   *Students, school administrators, staff and parents*   - Features of developed app:   - Gamified educational content,   - Prevention tool   - Resources   - Reporting features   **Phase II**   - Feedback on features from school community:   *Student Advisory Board*   - Liked the customizable avatar - Made several recommendations for app improvement   *Faculty and staff advisory board*   - Appreciated the contents and the features - Provided positive feedback on administrator dashboard - Appreciated customizability of school and community resources, push notification option, and suggested editing and viewing mode for dashboard - Discussed launching options - Prototype uSafeHS app and launching materials subsequently developed   **Phase III**  *Students*   - App usage: - Number of students who downloaded and used the app: 537 - Number of education modules completed: 1762 - Number of resources assessed: 51 - Number of Incident reports submitted and resolved: 8 - Feedback on app features: - Proportion who completed the module (of N = 8), liked the features, and said their knowledge had improved: 100% - Proportion (of N = 4 who used the feature) who thought they would likely use “Time to Leave” feature again: 75% - Proportion (of N = 5 who accessed app “Resources”) who used school resources: 100% - Proportion (of N = 5, who accessed resources) who used national resources: 80% | *Facilitators to app use*   - NR   *Barriers to app use*   - NR   *App limitations*   - NR | *Conclusions*   - Based on users’ perceptions, app can serve as a useful tool for high school students   *Recommendations*   - Students - Tutorial for first time users - Interactive app - “Short, informative and easy to learn and read” educational content - Additional information about improving relationship with self - Option to upload audio recording to “Time to Leave” feature - Avatar sharing options - More realistic and reliable modules, with current images - Including both call and text features in “Time to Leave” feature - Improved instructional message display and overall aesthetic features - School administrators and staff - Interactive app - Adding graphics - Digital screen for better awareness - Feature to facilitate difficult conversations among students - Tool to leave uncomfortable situations - After-hours reporting features   *Study limitations*   - Limitation in data interpretation at development phase - Small sample size |
| Self-harm/ Suicide prevention | | | | |
| BlueIce (UK) | | | | |
| - Grist et al., 2018 [13] - Stallard et al., 2018 [14] | - To assess the acceptability, use, and safety of BlueIce [13] - To assess the effects of BlueIce on self-harm and psychological functioning in young people [14] | - Acceptability: - Proportion who wanted to use the app after trialing it for 2 weeks: 93% - Proportion who wanted to keep the app after 12 weeks: 88% [14] - Many participants though that adding a game feature would be beneficial - Most helpful app features: - Wide range of techniques available - “Having an outlet for their emotions” - “Quick access to emergency numbers” [13] - Implemented recommendations: - Additional options added to mood checker, such as one that allowed users to express themselves in their own words [13] - Colors added to mood wheel options (range: red (really sad) to green (really happy) [13] - Text option (in addition to the regular calling option) [13] - ‘Other” option that allowed users to express their feelings - All participants would recommend the app to others [13] - Usability: - Higher rate of app use at beginning of study. Reasons for this include: - Improvements in mental health outcomes   - - Use of app only when individuals felt low [13]     - Simple and easy to use [13] - Accessible, easy to navigate, and not burdensome [13]Popular app features:   - Mood diary   - Mood lifter [13] - Main benefits of the app:   - Tracking and recognizing mood patterns   - Identifying triggers for negative emotions [13] - Self-monitoring helped with:   - Dealing with “less positive” feeling   - Developing emotional awareness [13] - Safety - Helped with prevention of urges to self-harm - Prevented l from continued self-harm after an initial instance of self-harming - No adverse events - Desired to keep the app beyond the study [13] - Depression   *App users*   - Statistically significant decrease in scores (P=.02) [14]   *Parents*   - No statistically significantly difference [14] - Anxiety   *App users*   - Statistically significant reduction in overall scores across all five sub-scales (panic disorder, separation anxiety disorder, generalized anxiety disorder, and social anxiety disorder (P ranged from .07 – .02) [14] - Behavior   *App users*   - Non-significant reduction in total score - Statistically significant reduction in emotional symptom sub-scale score only (P=.001)   *Parents*   - Statistically significant reduction in overall score - Statistically significant decrease in emotional subscale and peer relationship subscale scores [14] - Self-harm   *App users*   - Participants who did not self-harm in the 4 weeks prior to the study maintained their non-self-harming status - Proportion who stopped their self-harm behavior: 15% - Proportion who self-harmed less frequently: 58% - Proportion with no reduction in self-harming behavior: 27% - Number of incidents of self-harm prevented during study: 308 - Potential reduction in healthcare cost due to reduction in self-harming incidence which would likely have required contact with health care services - Reasons for no improvement in self-harm (N = 7): - App perceived as repetitive - App not perceived as helpful - App users did not believe that app could help them overcome their difficulties - Annoyance from being asked about their feelings - Preference of face-to face engagement - Lack of motivation to use app due to not being ready to stop self-harming [13] | *Facilitators to app use*   - Private, discreet, and confidential nature of the app - Mood diary [13]   *Barriers to app use*   - Only available on Android - Certain circumstances (e.g. school, spending time with family)   *App limitations*   - limited effectiveness in preventing self-harm [13, 14] | *Conclusions*   - Based on users’ perceptions, app is acceptable, easy to use, and safe for young people who self-harm [13, 14] - Based on high level of involvement via referrals, app is acceptable to clinicians - App appears to be useful for preventing self-harm in young people, in conjunction with traditional face-to-face therapy [13, 14]   *Recommendations*   - Further personalization of app by adding different colors - Adding a game to the app [13]   Limitations   - No independent control group - Small sample size - Self-selection bias: participants actively elected to try the app, and would likely have had more positive perceptions of the app than a randomly selected sample - Reporting bias - Recall bias - It cannot be determined if the improved psychological symptoms were due to BlueIce use, face-to-face interventions, or synergistic effects [13, 14] |
| iBobbly (Australia) | | | | |
| - Tighe et al., 2020 [15] | - To determine the pilot usage and acceptability of the iBobbly suicide prevention app | *App users*   - General internet use - Time engaged online: - Typical time spent online: 4 hours per day; range: 30 minutes – 10 hours - Time spent online during weekends: mean: 3.36 hours per day; range: 0 – 9 hours - Most popular times for online activity: afternoons and evenings (from 3 pm onwards) - Least common time for online use: prior to noon - All participants engaged in online activities after 11 pm at least 1 day per week - Proportion who went online after 11 pm at least 3 days per week: 31% - Devices of choice for online access: - Proportion who used a smartphone daily: 77% (10/13) - Proportion who used a tablet: 54% (7/13) - Proportion who used a laptop: 23% (3/13) - Proportion who used gaming technologies or consoles: 15% (2/13) - Proportion who used a desktop computer: 0 - Proportion who reported that they would miss their smartphone more than any other technology if they no longer had access to it: 67% (9/13) - Proportion who reported that they would miss their television most: 23% (3/13) [15] - Place/device used for internet access: - Smartphone or tablet: 85% (11/13) - At work or in a public place (library, internet café, etc.): 15% (2/13) - Online activities: - Engaging on social networking sites or apps such as Facebook: 85% (11/13) - Email: 69% (9/13) - Watching or downloading video clips or movies: 61% (8/13) - Accessing health information or gaming: 46% (6/13) - App usage: - Average total time spent using app: 73.7 minutes; range: 8 minutes - 5 hours and 42 minutes - Proportion who used the app for more than 1 hour (37.5% (15/40) - Average number of visits to the app: 12.4; range: 1 – 43) - Average duration of a session on the app: 5.94 minutes - Proportion who completed all 6 activities on the app: 85% (34/40) - Participants with higher psychological needs used the app more frequently - Users’ perception: - Acceptability: - Many participants considered the app to be acceptable - Proportion who would recommend app to others: 100% (13/13) - Proportion who would participate in a similar trial again: 92% (12/13) - Cultural appropriateness: - Reported to be culturally safe and relevant - Assisted in seeking future help - Therapeutic effectiveness: - Created distractions and reduced the distress of users - Gave participants “language to their experiences” - Helped users identify potentially harmful behaviors including suicidality, and sometimes changed their perspectives and presented coping strategies that prevented them from self-harm - Provided an interruption for users and gave them space to improve their mental state and decision making - Helped users improve self-awareness and interpersonal communications - Suicidal ideation, depression, and psychological distress: - No significant correlation between app usage time and any of the 3 outcomes analyzed (suicidal ideation, depression, and psychological distress), but all associations were positive (improved outcomes) | *Facilitators to app use*   - Accessibility and privacy - Mobility: consistent and appropriate support in time of need regardless of users’ location - No shame and embarrassment as compared to speaking with family members or healthcare professionals   *Barriers to app use*   - NR   *App limitations*   - Repetitiveness: made users feel frustrated (but not suicidal) - Lack of advance tools that could allow users to move to more advanced set of questions; basic; not challenging - Risk of invoking negative feelings with no follow-up support | *Conclusions*   - Aboriginal and Torres Strait Islander youth have a relatively high level of engagement with the mobile app for suicide prevention and social and emotional wellbeing - Based on users’ perceptions, app was acceptable and culturally appropriate for Aboriginal and Torres Strait Islander youth and their community members   *Recommendations*   - Additional content to iBobbly to make it longer and more advanced to mitigate against user frustration due to repetitiveness   *Study limitations*   - Small sample size - Short follow-up period |
| - Tighe et al., 2017 [16] | - To assess the impact of iBobbly app on suicidal ideation, depression, psychological distress and impulsivity in Aboriginal and Torres Strait Islander youth - To pilot the implementation of an RCT in Aboriginal and Torres Strait Islander communities with a focus on suicide prevention | *App users*   - App use: - Proportion who completed all 6 activities: 85% - Proportion who completed 5/6 activities: 2.5% - Proportion who completed 2/6 activities: 12.5% - Users’ perception: - App was acceptable and culturally appropriate to Aboriginal and Torres Strait Islander youth (as indicated by heir willingness to recommend it to others in their networks) - Suicidal ideation: - Non-statistically significant difference in reduction in suicide ideation between iBobbly arm (30% reduction) and control arm (17% reduction) - Statistically significant pre-post reduction in intervention arm (P=.020) - Depression: - Statistically significant reduction in depression in iBobbly arm (42%) compared to control arm (17%) - Statistically significant pre-post reduction in intervention arm (P=.007) - Psychological distress: - Statistically significant reduction in psychological distress in iBobbly arm (28%) compared to control arm (12%) - Statistically significant pre-post reduction in intervention arm (P=.018) - Impulsivity: - No differences in impulsivity between iBobbly arm and control arm after 6 weeks of app use | *Facilitators to app use*   - All text accompanied by gender-matched audio, which assisted people with limited literacy   *Barriers to app use*   - Technical/ connectivity failure of mobile device   *App limitations*   - NR | *Conclusions*   - App use reduces distress and depression but does not significantly impact suicide ideation or impulsivity - App is a feasible and acceptable tool for lowering symptoms for mental   health disorders in remote communities  *Recommendations*   - NR   *Study limitations*   - Small sample size, particularly for participants without suicide ideation at baseline, which reduced the chances of obtaining significant results in this outcome - Short follow-up period - Difficult to determine the therapeutic effectiveness of app use in such studies |

**References**

1. Sheoran B, Silva CL, Lykens JE, Gamedze L, Williams S, Ford JV, et al. YTH StreetConnect: Development and Usability of a Mobile App for Homeless and Unstably Housed Youth. JMIR mHealth and uHealth. 2016;4(3):e82. [doi: [10.2196/mhealth.5168](https://doi.org/10.2196/mhealth.5168)]

2. Blayney JA, Jenzer T, Read JP, Livingston JA, Testa M. Enlisting friends to reduce sexual victimization risk: There's an app for that… but nobody uses it. J Am Coll Health. 2018;66(8):767-73. [doi: [10.1080/07448481.2018.1446439](https://doi.org/10.1080/07448481.2018.1446439)]

3. Navarro-Pérez JJ, Oliver A, Carbonell Á, Schneider BH. Effectiveness of a mobile app intervention to prevent dating violence in residential child care. Psychosoc Interv. 2020;29(2):59-66. [doi: [10.5093/pi2020a3](https://doi.org/10.5093/pi2020a3)]

4. Navarro-Pérez J-J, Carbonell Á, Oliver A. The effectiveness of a psycho-educational app to reduce sexist attitudes in adolescents. Rev Psicodidáct (English ed.). 2019;24(1):9-16. [doi: [10.1016/j.psicod.2018.07.002](https://doi.org/10.1016/j.psicod.2018.07.002)]

5. Glass N, Clough A, Messing J, Bloom T, Brown M, Eden K, et al. Longitudinal Impact of the myPlan App on Health and Safety Among College Women Experiencing Partner Violence. J Interpers Violence. 2022;37(13-14). [doi: [10.1177/0886260521991880](https://doi.org/10.1177/0886260521991880)]

6. Debnam K, Kumodzi T. Adolescent Perceptions of an Interactive Mobile Application to Respond to Teen Dating Violence. J Interpers Violence. 2021;36(13-14). [doi: [10.1177/0886260518821455](https://doi.org/10.1177/0886260518821455)]

7. Alhusen J, Bloom T, Clough A, Glass N. Development of the MyPlan Safety Decision App with Friends of College Women in Abusive Dating Relationships. J Technol Hum Serv. 2015;33(3):263-82. [doi: [10.1080/15228835.2015.1037414](https://doi.org/10.1080/15228835.2015.1037414)]

8. Lindsay M, Messing JT, Thaller J, Baldwin A, Clough A, Bloom T, et al. Survivor Feedback on a Safety Decision Aid Smartphone Application for College-Age Women in Abusive Relationships. J Technol Hum Serv. 2013;31(4):368-88. [doi: [10.1080/15228835.2013.861784](https://doi.org/10.1080/15228835.2013.861784)]

9. Potter SJ, Moschella EA, Demers JM, Lynch M. Using Mobile Technology to Enhance College Sexual Violence Response, Prevention, and Risk Reduction Efforts. J Technol Hum Serv. 2022;40(1):25-46. [doi: [10.1080/15228835.2021.1929665](https://doi.org/10.1080/15228835.2021.1929665)]

10. Potter SJ, Moschella EA, Smith D, Draper N. Exploring the Usage of a Violence Prevention and Response App Among Community College Students. Health Educ Behav. 2020;47(1_suppl):44S-53S. [doi: [10.1177/1090198120910995](https://doi.org/10.1177/1090198120910995)]

11. Ouellet-Morin I, Robitaille M-P. Stronger than Bullying, a mobile application for victims of bullying: Development and initial steps toward validation. In: Campbell M, Bauman S, editors. Reducing Cyberbullying in Schools. Academic Press; 2018. p. 159-74.

12. Potter SJ, Moschella-Smith EA, Lynch M. Building a high school violence prevention app to educate and protect students. J Res Technol Educ. 2022:1-19. [doi: [10.1080/15391523.2022.2110336](https://doi.org/10.1080/15391523.2022.2110336)]

13. Grist R, Porter J, Stallard P. Acceptability, Use, and Safety of a Mobile Phone App (BlueIce) for Young People Who Self-Harm: Qualitative Study of Service Users’ Experience. JMIR Mental Health. 2018;5(1):e16. [doi: [10.2196/mental.8779](https://doi.org/10.2196/mental.8779)]

14. Stallard P, Porter J, Grist R. A Smartphone App (BlueIce) for Young People Who Self-Harm: Open Phase 1 Pre-Post Trial. JMIR mHealth and uHealth. 2018;6(1):e32. [doi: [10.2196/mhealth.8917](https://doi.org/10.2196/mhealth.8917)]

15. Tighe J, Shand F, Mckay K, Mcalister T-J, Mackinnon A, Christensen H. Usage and Acceptability of the iBobbly App: Pilot Trial for Suicide Prevention in Aboriginal and Torres Strait Islander Youth. JMIR Mental Health. 2020;7(12):e14296. [doi: [10.2196/14296](https://doi.org/10.2196/14296)]

16. Tighe J, Shand F, Ridani R, Mackinnon A, De La Mata N, Christensen H. Ibobbly mobile health intervention for suicide prevention in Australian Indigenous youth: a pilot randomised controlled trial. BMJ Open. 2017;7(1):e013518. [doi: [10.1136/bmjopen-2016-013518](https://doi.org/10.1136/bmjopen-2016-013518)]
